# A whole-slide foundation model for digital pathology from real-world data

**DOI:** 10.1038/s41586-024-07441-w

**Published:** 2024-05-22

**Authors:** Hanwen Xu, Naoto Usuyama, Jaspreet Bagga, Sheng Zhang, Rajesh Rao, Tristan Naumann, Cliff Wong, Zelalem Gero, Javier González, Yu Gu, Yanbo Xu, Mu Wei, Wenhui Wang, Shuming Ma, Furu Wei, Jianwei Yang, Chunyuan Li, Jianfeng Gao, Jaylen Rosemon, Tucker Bower, Soohee Lee, Roshanthi Weerasinghe, Bill J. Wright, Ari Robicsek, Brian Piening, Carlo Bifulco, Sheng Wang, Hoifung Poon

**Affiliations:** 1 Microsoft Research, Redmond, WA USA; 2 Paul G. Allen School of Computer Science and Engineering, University of Washington, Seattle, WA USA; 3 Providence Genomics, Portland, OR USA; 4 Providence Research Network, Renton, WA USA; 5 Earle A. Chiles Research Institute, Providence Cancer Institute, Portland, OR USA; 6 Department of Surgery, University of Washington, Seattle, WA USA

**Keywords:** Pathology, Cancer imaging

## Abstract

Digital pathology poses unique computational challenges, as a standard gigapixel slide may comprise tens of thousands of image tiles^1–3^. Prior models have often resorted to subsampling a small portion of tiles for each slide, thus missing the important slide-level context^4^. Here we present Prov-GigaPath, a whole-slide pathology foundation model pretrained on 1.3 billion 256 × 256 pathology image tiles in 171,189 whole slides from Providence, a large US health network comprising 28 cancer centres. The slides originated from more than 30,000 patients covering 31 major tissue types. To pretrain Prov-GigaPath, we propose GigaPath, a novel vision transformer architecture for pretraining gigapixel pathology slides. To scale GigaPath for slide-level learning with tens of thousands of image tiles, GigaPath adapts the newly developed LongNet^5^ method to digital pathology. To evaluate Prov-GigaPath, we construct a digital pathology benchmark comprising 9 cancer subtyping tasks and 17 pathomics tasks, using both Providence and TCGA data^6^. With large-scale pretraining and ultra-large-context modelling, Prov-GigaPath attains state-of-the-art performance on 25 out of 26 tasks, with significant improvement over the second-best method on 18 tasks. We further demonstrate the potential of Prov-GigaPath on vision–language pretraining for pathology^7,8^ by incorporating the pathology reports. In sum, Prov-GigaPath is an open-weight foundation model that achieves state-of-the-art performance on various digital pathology tasks, demonstrating the importance of real-world data and whole-slide modelling.

## Abstract (web summary)

Prov-GigaPath, a whole-slide pathology foundation model pretrained on a large dataset containing around 1.3 billion pathology images, attains state-of-the-art performance in cancer classification and pathomics tasks.

## Main

Computational pathology has the potential to transform cancer diagnostics by empowering diverse clinical applications, including cancer subtyping^2,9,10^, cancer staging^1,11–13^, diagnostic prediction^14–17^ and prognostic prediction^18–23^. Despite the encouraging performance of existing computational approaches, these are often developed for a specific application and require a large amount of annotated data for supervised learning. Data annotation is expensive and time-consuming and has emerged as an important bottleneck for computational pathology. Recently, self-supervised learning has shown promising results in leveraging unlabelled data to pretrain a foundation model, which can substantially reduce the demand for task-specific annotations^24–28^. Owing to their strong generalizability, foundation models have been developed for biomedical domains where labelled data are scarce but unlabelled data are abundant, a situation that aptly describes computational pathology^29–33^.

There are three major challenges that hinder the development and use of pathology foundation models for real-world clinical applications. First, publicly available pathology data are relatively scarce and of varying quality, which limits the performance of foundation models pretrained on such data. For example, existing pathology foundation models were mainly pretrained on whole-slide images (WSIs) from The Cancer Genome Atlas (TCGA), an expert-curated dataset comprising approximately 30,000 slides and 208 million image tiles. Although they are a tremendous resource, TCGA data might not be sufficiently large to fully address the challenges around real-world digital pathology in clinical practice, such as heterogeneity and noise artefacts^34^, leading to a substantial performance drop when using TCGA-based predictive models and biomarkers on out-of-distribution samples. Second, it remains challenging to design a model architecture that can effectively capture both local patterns in individual tiles and global patterns across whole slides^35–39^. Existing models often treat each image tile as an independent sample and formulate slide-level modelling as multiple instance learning^4,40–43^, thus limiting their ability to model complex global patterns in gigapixel whole slides. A notable exception is Hierarchical Image Pyramid Transformer (HIPT), which explores hierarchical self-attention over the tiles^35^. Third, in the rare cases in which pretraining has been conducted on large-scale real-world patient data, the resulting foundation models are typically not accessible to the public, thus limiting their broader applicability in clinical research and applications.

We have developed Prov-GigaPath, an open-weight pathology foundation model, to address these three challenges (Supplementary Table 1). First, Prov-GigaPath is pretrained on Prov-Path, a large digital pathology dataset from the Providence health network across 28 cancer centres. Prov-Path contains 1,384,860,229 image tiles from 171,189 haematoxylin and eosin (H&E)-stained and immunohistochemistry pathology slides, which originated from biopsies and resections in more than 30,000 patients, covering 31 major tissue types. Prov-Path is more than five times larger than TCGA in terms of the number of image tiles and more than two times larger than TCGA in terms of the number of patients. Our pretraining leverages all 1.3 billion image tiles, which, to our knowledge, constitutes the largest pretraining effort to date. These large, diverse, real-world data serves as the foundation for pretraining Prov-GigaPath. Prov-Path also encompasses a hierarchy of valuable information, including histopathology findings, cancer staging, genomic mutation profiles, along with the associated pathology reports.

Second, to capture both local and global patterns across the entire slide, we propose GigaPath, a novel vision transformer for pretraining large pathology foundation models on gigapixel pathology slides. The key idea is to embed image tiles as visual tokens, thus turning a slide into a long sequence of tokens. Transformer^44^ is a powerful neural architecture for sequence modelling by distilling arbitrary complex patterns among the tokens. However, we cannot directly apply a conventional vision transformer to digital pathology, as a pathology slide may contain tens of thousands of tiles (as many as 70,121 in the Providence data) and computation with self-attention in transformer grows quadratically in the sequence length. To address this problem, we leverage dilated self-attention by adapting our recently developed LongNet method^5^. Pretraining starts with image-level self-supervised learning using DINOv2^24^ with standard vision transformer, followed by whole-slide-level self-supervised learning using masked autoencoder^45^ with LongNet.

Finally, to accelerate research progress in digital pathology, we make Prov-GigaPath fully open-weight, including source code and pretrained model weights.

To systematically investigate the effectiveness of Prov-GigaPath as a pathology foundation model in real-world scenarios, we established a comprehensive digital pathology benchmark spanning 26 prediction tasks such as pathomics and cancer subtyping, using data from both Providence and TCGA. We compare Prov-GigaPath against the state-of-the-art pathology foundation models that are publicly available, including HIPT^35^, CtransPath^41^ and REMEDIS^42^. Combining large-scale pretraining and ultra-large-context modelling, Prov-GigaPath attains state-of-the-art performance on 25 out of 26 tasks, with significant improvement over the second-best method in 18 tasks (Supplementary Table 2). For example, on the TCGA dataset for EGFR mutation prediction, Prov-GigaPath attained an improvement of 23.5% in AUROC and 66.4% in AUPRC compared with the second-best model, REMEDIS. This is particularly remarkable as REMEDIS was pretrained on TCGA data whereas Prov-GigaPath was not. For cancer subtyping, Prov-GigaPath outperforms all other models in all nine cancer types, with significant improvement over the second-best method in six cancer types. This bodes well for its broad applicability across cancer types. Finally, we explore vision–language pretraining by leveraging the associated pathology report for each slide to continue pretraining Prov-GigaPath with vision–language contrastive learning. We showed that the resulting Prov-GigaPath exhibits state-of-the-art capability in standard vision–language modelling tasks such as zero-shot subtyping and mutation prediction, illustrating its potential for multimodal integrative data analysis. In sum, Prov-GigaPath demonstrates the possibility to assist clinical diagnostics and decision support using large-scale machine learning models.

## Overview of Prov-GigaPath

**Fig. 1:**
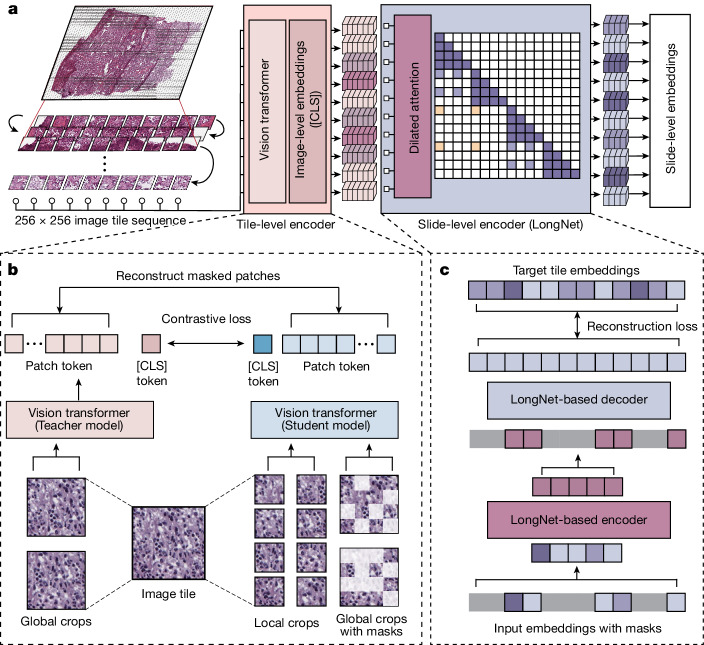
Overview of Prov-GigaPath*.* **a**, Flow chart showing the model architecture of Prov-GigaPath. Prov-GigaPath first serializes each input WSI into a sequence of 256 × 256 image tiles in row-major order and uses an image tile-level encoder to convert each image tile into a visual embedding. Then Prov-GigaPath applies a slide-level encoder based on the LongNet architecture to generate contextualized embeddings, which can serve as the basis for various downstream applications. **b**, Image tile-level pretraining using DINOv2. **c**, Slide-level pretraining with LongNet using masked autoencoder. [CLS] is the classification token.

Prov-GigaPath takes the image tiles in a pathology slide as input and outputs the slide-level embeddings that can be used as features for diverse clinical applications (Fig. 1a). Prov-GigaPath excels in long-context modelling of gigapixel pathology slides, by distilling varied local pathological structures and integrating global signatures across the whole slide. Prov-GigaPath consists of a tile encoder for capturing local features and a slide encoder for capturing global features. The tile encoder individually projects all tiles into compact embeddings. The slide encoder then inputs the sequence of tile embeddings and generates contextualized embeddings taking into account the entire sequence using a transformer. The tile encoder is pretrained using DINOv2, the state-of-the-art image self-supervised learning framework^24^. The slide encoder combines masked autoencoder pretraining with LongNet^5^, our recently developed method for ultra long-sequence modelling. In downstream tasks, the output of the slide encoder is aggregated using a simple softmax attention layer. Prov-GigaPath is a general pretraining method for high-resolution imaging data, which can potentially be extended to other biomedical problems, including the analysis of large 2D and 3D images and videos. We pretrained Prov-GigaPath on the large and diverse real-world data in Prov-Path. Given a downstream task, the pretrained Prov-GigaPath is fine-tuned using task-specific training data, as standard in the use of a foundation model. The resulting task-specific model can then be evaluated on the test data for the given task. Prov-GigaPath attained significant improvements compared to prior state-of-the-art public pathology foundation models across 17 pathomics tasks and 9 subtyping tasks. Our pretraining dataset Prov-Path consists of 1,384,860,229 256 × 256 image tiles in 171,189 H&E-stained and immunohistochemistry pathology slides, which stem from biopsies and resections of 31 major tissue types in over 30,000 patients (Supplementary Figs. 1–3). We summarize the demographics, including the distribution of sex, age and race in Supplementary Tables 3–5 and the mutation rates in Supplementary Table 6.

## Prov-GigaPath improves mutation prediction

**Fig. 2:**
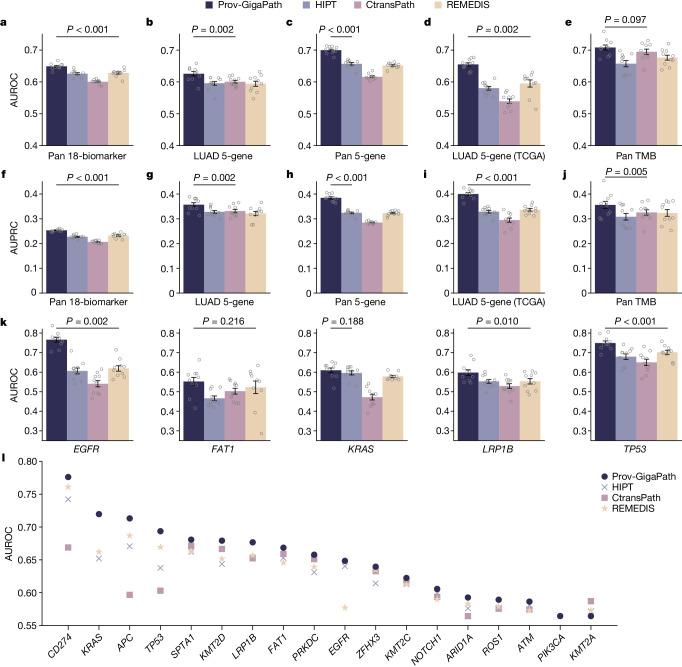
Gene mutation prediction. **a**−**j**, Bar plots comparing the AUROC and AUPRC scores of Prov-GigaPath and competing methods on pan-cancer 18-biomarker (**a**,**f**), LUAD-specific 5-gene mutation prediction (**b**,**g**), pan-cancer 5-gene mutation prediction (**c**,**h**), LUAD-specific 5-gene mutation prediction on TCGA (**d**,**i**) and pan-cancer TMB prediction (**e,j**). **k**, Bar plot showing AUROC for each gene on LUAD-specific five-gene mutation prediction on TCGA. **a**−**k**, Data are mean ± s.e.m. across *n* = 10 independent experiments. The listed *P* value indicates the significance for Prov-GigaPath outperforming the best comparison approach, with one-sided Wilcoxon test. **l**, Comparison of AUROC scores for individual biomarkers in pan-cancer 18-biomarker predictions.

**Extended Data Fig. 1:**
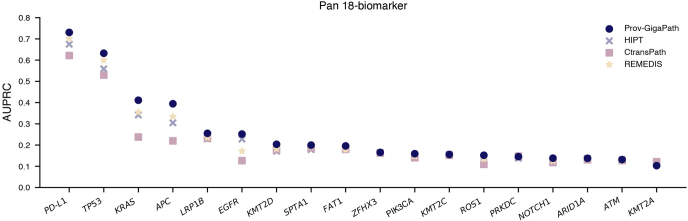
Comparison on Pan-cancer 18-biomarker prediction. Bar plot showing the AUPRC score for each biomarker on the 18-biomarker prediction by *Prov-GigaPath* and competing methods.

**Extended Data Fig. 2:**
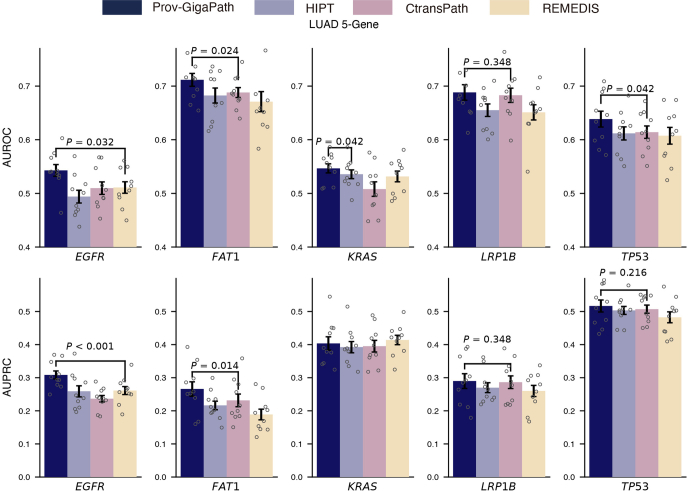
Comparison on LUAD 5-gene mutation prediction. Bar plots showing AUROC and AUPRC scores for predicting each gene mutation on LUAD 5-gene mutation prediction. The error bars show the standard error across n = 10 independent experiments and the bar centre shows the mean value. The listed *p*-value indicates the significance level that *Prov-GigaPath* outperforms the best comparison approach, with one-sided Wilcoxon test.

**Extended Data Fig. 3:**
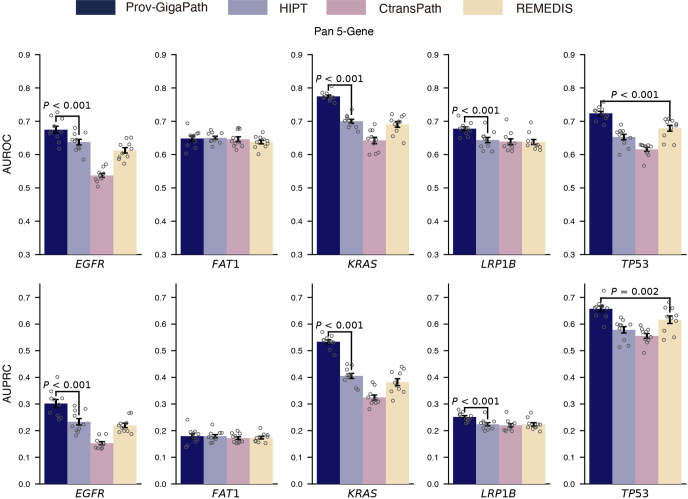
Comparison on Pan-cancer 5-gene mutation prediction. Bar plots showing AUROC and AUPRC scores for predicting each gene mutation on Pan-cancer 5-gene mutation prediction. The error bars show the standard error across n = 10 independent experiments and the bar centre shows the mean value. The listed *p*-value indicates the significance level that *Prov-GigaPath* outperforms the best comparison approach, with one-sided Wilcoxon test.

**Extended Data Fig. 4:**
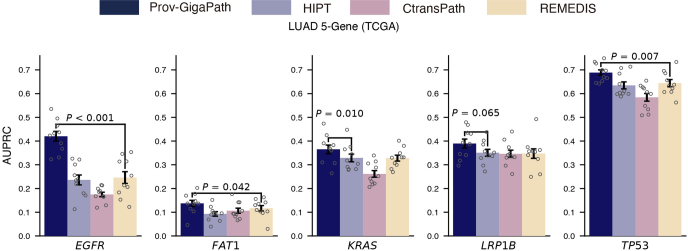
Comparison on LUAD 5-gene mutation prediction in TCGA. Bar plots showing AUPRC scores for predicting each gene mutation on LUAD 5-gene mutation prediction in TCGA. The error bars show the standard error across n = 10 independent experiments and the bar centre shows the mean value. The listed *p*-value indicates the significance level that *Prov-GigaPath* outperforms the best comparison approach, with one-sided Wilcoxon test.

A variety of function-altering somatic gene mutations underlie cancer progression and development, and thus may have utility in both cancer diagnostics and prognostics. Although the cost of sequencing has dropped substantially, there are still critical healthcare gaps in terms of access to tumour sequencing worldwide. Therefore, predicting tumour mutations from pathology images may help to inform treatment selection and increase personalized medicine utilization^17^. We compared Prov-GigaPath with competing methods on five-gene mutation prediction benchmarks (Fig. 2 and Extended Data Figs. 1–4) by formulating this task as an image classification task. First, we examined the prediction of 18 biomarkers that are most frequently mutated in a pan-cancer setting (Fig. 2a,f,l and Extended Data Fig. 1). Prov-GigaPath achieved 3.3% macro-area under the receiver operator characteristic (AUROC) improvement and 8.9% macro-area under the precision-recall curve (AUPRC) improvement across these 18 biomarkers compared with the best competing method. Given known associations between specific tumour mutations and overall tumour composition and morphology, we attribute this improvement to the ability of LongNet to effectively capture the global image patterns. Next, we focused on lung adenocarcinoma (LUAD), which is one of the most widely studied cancer types for image-based mutation prediction (Fig. 2b,g and Extended Data Fig. 2). We focused on five genes (*EGFR*, *FAT1*, *KRAS*, *TP53* and *LRP1B*) that are closely related to LUAD diagnosis and treatment in the literature^46–48^. Prov-GigaPath demonstrated the best performance by achieving an average macro-AUROC of 0.626, surpassing all competing approaches (*P* value < 0.01). On the pan-cancer analysis, Prov-GigaPath also outperformed the best competing methods on these 5 genes with 6.5% macro-AUROC improvement and 18.7% AUPRC improvement (Fig. 2c,h and Extended Data Fig. 3).

**Extended Data Fig. 5:**
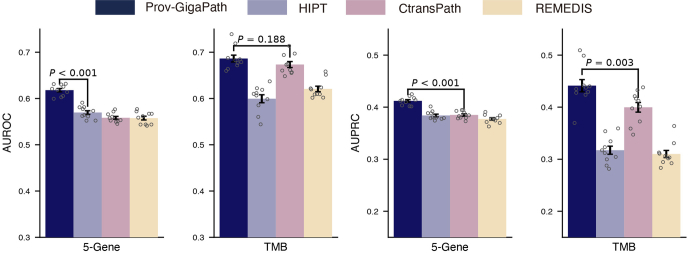
Comparison on mutation prediction on new colorectal patients. Bar plots showing AUROC and AUPRC scores for predicting 5-gene mutation and TMB status on new patients from Providence. The error bars show the standard error across n = 10 independent experiments and the bar centre shows the mean value. The listed *p*-value indicates the significance level that *Prov-GigaPath* outperforms the best comparison approach, with one-sided Wilcoxon test.

We also conducted head-to-head comparison of all approaches on TCGA data to examine the generalizability of Prov-GigaPath. We again used LUAD-specific five-gene mutation prediction as a key evaluation task (Fig. 2d,i and Extended Data Fig. 4). We observed similar advantage of Prov-GigaPath over the competing methods. This is all the more remarkable given that the competing methods^35,41,42^ were all pretrained on TCGA. To further test the generalizability of Prov-GigaPath, we collected a new cohort of 403 patients with colorectal cancer from Providence. These data were collected after March 2023, whereas all data used for pretraining Prov-GigaPath were collected before March 2023. We found that Prov-GigaPath again outperformed competing methods on this cohort. We also noted that the performance was not significantly different from that on previous data from patients with colorectal cancer (Extended Data Fig. 5). Finally, we examined the prediction of overall tumour mutation burden (TMB), a predictive biomarker in solid tumours that is particularly relevant for immunotherapy. Prov-GigaPath achieved the best performance with an average AUROC of 0.708, with significant improvement over the second-best method (Fig. 2e,j).

**Extended Data Fig. 6:**
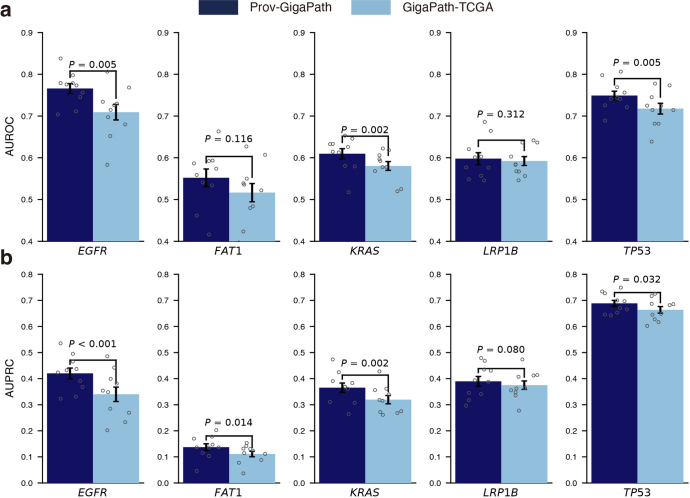
Comparison between pretraining the same model using *Prov-Path* and TCGA. **a-b**, Bar plots showing the AUROC (**a**) and AURPC (**b**) on LUAD 5-gene mutation prediction in TCGA using models trained on *Prov-Path* and TCGA. *Prov-GigaPath* is *GigaPath* trained on *Prov-Path*. GigaPath-TCGA is *GigaPath* trained on TCGA. The error bars show the standard error across n = 10 independent experiments and the bar centre shows the mean value. The listed *p*-value indicates the significance level that *Prov-GigaPath* outperforms GigaPath-TCGA, with one-sided Wilcoxon test.

**Extended Data Fig. 7:**
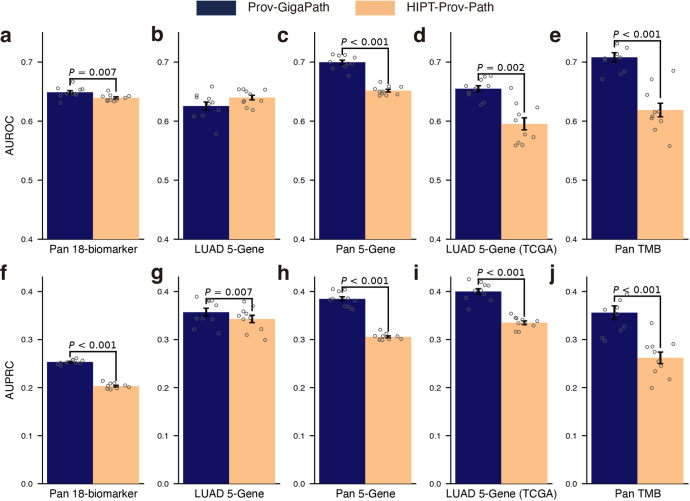
Comparison between *GigaPath* trained using *Prov-Path* and HIPT trained using *Prov-Path* on mutation prediction. **a-j**: Bar plots showing the AUROC (**a-e**) and AURPC (**f-j**) of mutation prediction tasks by *Prov-GigaPath* and HIPT-Prov-Path. HIPT-Prov-Path indicates HIPT pretrained on *Prov-Path*. The error bars show the standard error across n = 10 independent experiments and the bar centre shows the mean value. The listed *p*-value indicates the significance level that *Prov-GigaPath* outperforms the HIPT-Prov-Path, with one-sided Wilcoxon test.

**Extended Data Fig. 8:**
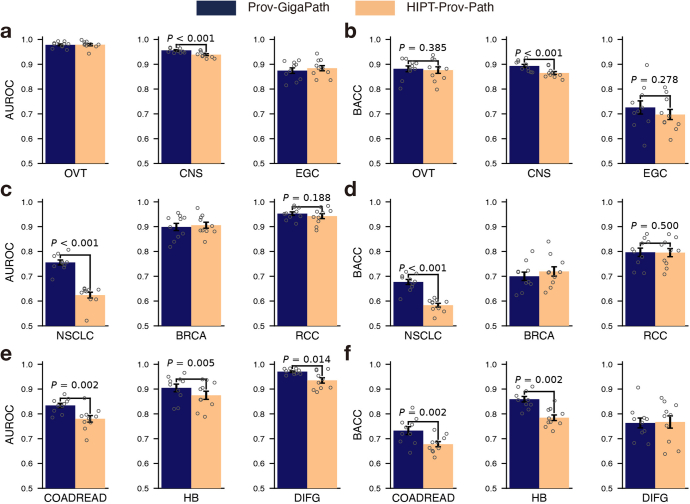
Comparison between *GigaPath* trained using *Prov-Path* and HIPT trained using *Prov-Path* on cancer subtyping. **a-f**, Bar plots showing the AUROC (**a,c,e**) and BACC (**b,d,f**) of cancer subtyping tasks by *Prov-GigaPath* and HIPT-Prov-Path. HIPT-Prov-Path indicates HIPT pretrained on *Prov-Path*. The error bars show the standard error across n = 10 independent experiments and the bar centre shows the mean value. The listed *p*-value indicates the significance level that *Prov-GigaPath* outperforms the HIPT-Prov-Path, with one-sided Wilcoxon test.

We observed that GigaPath pretrained on Prov-Path achieves a substantial improvement against the same model architecture pretrained on TCGA data when tested on LUAD-specific five-gene mutation in TCGA, indicating the high quality of Prov-Path (Extended Data Fig. 6). We further found that GigaPath outperformed HIPT when both are trained on Prov-Path, indicating that the effectiveness of GigaPath framework (Extended Data Figs. 7 and 8). To further assess the pretraining strategy of our method, we observed that pretraining using DINOv2 is better than pretraining using a contrastive-learning-based approach SimCLR^26^ and masked autoencoders^45^ (Supplementary Fig. 4), demonstrating the effectiveness of our pretraining strategy. Prov-GigaPath also outperformed a supervised learning approach that utilizes an ImageNet-trained model, necessitating our self-supervised learning framework (Supplementary Fig. 4).

Overall, Prov-GigaPath demonstrated clear performance gains on various pathomics tasks over prior state-of-the-art pathology foundation models. We hypothesize that such significant improvement reflects the differentiation advantage in our whole-slide modelling.

## Prov-GigaPath improves cancer subtyping

**Fig. 3:**
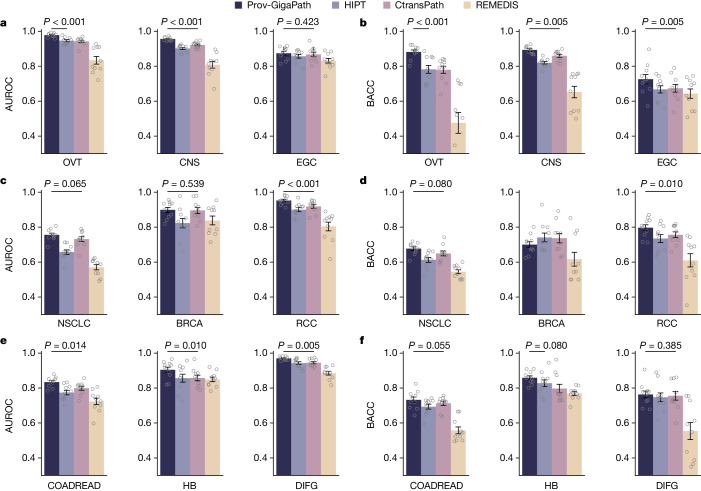
Comparison on cancer subtyping. **a**–**f**, Bar plots comparing cancer subtyping performance in terms of AUROC (**a**,**c**,**e**) and balanced accuracy (**b**,**d**,**f**) on nine cancer types. Data are mean ± s.e.m. across *n* = 10 independent experiments. The listed *P* value indicates the significance for Prov-GigaPath outperforming the best comparison approach, with one-sided Wilcoxon test. BACC, balanced accuracy. BRCA, breast invasive carcinoma; CNS, central nervous system; COADREAD, colorectal adenocarcinoma; DIFG, diffuse intrinsic pontine glioma; EGC, early gastric cancer; HB, hepatobiliary; NSCLC, non-small cell lung cancer; OVT, ovarian cancer; RCC, renal cell cancer.

Given the overall utility of pathology images in assigning tumour subtypes^2,9,10,49^, we next examined whether Prov-GigaPath can accurately predict cancer subtypes from images. We evaluated our method on subtyping for nine major cancer types in Prov-Path (Fig. 3). Prov-GigaPath outperformed all competing approaches on all nine cancer types and achieved significant improvements compared with the second-best method on six cancer types, indicating that our tile encoder and slide encoder work synergistically to extract meaningful features for differentiating minute pathological patterns. A key difference between HIPT and Prov-GigaPath is the aggregation layer over image tile embeddings. The substantial improvement of Prov-GigaPath over HIPT demonstrates the promise in using LongNet for efficient and effective aggregation of the ultra-large collection of image tiles in a whole slide.

Finally, we conducted ablation studies to systematically assess how each component of Prov-GigaPath contributes to its performance in cancer subtyping (Supplementary Fig. 5). To examine the importance of LongNet pretraining, we replaced the LongNet encoder pretrained on Prov-Path with a randomly initialized model. We observed a substantial performance decrease in average AUROC from 0.903 to 0.886 (*P* value < 2.0 × 10^−3^), indicating that pretraining our LongNet encoder could better capture the slide-level cancer heterogeneity. We observed that freezing and unfreezing the LongNet encoder achieved comparable performance on cancer subtyping tasks. This suggests that our pretraining approach can effectively learn high-quality representations, reducing the need for additional fine-tuning of LongNet. To verify the superiority of using the LongNet encoder to aggregate image patterns across the whole slide, we then tested one alternative by removing LongNet and only aggregating through the attention-based deep multiple instance learning (ABMIL) layer. On average, the ABMIL layer cannot achieve a similar performance to LongNet for slide encoder (*P* value < 0.012), confirming the necessity of modelling long-range dependencies in pathology slides.

## Slide-level vision–language alignment

**Fig. 4:**
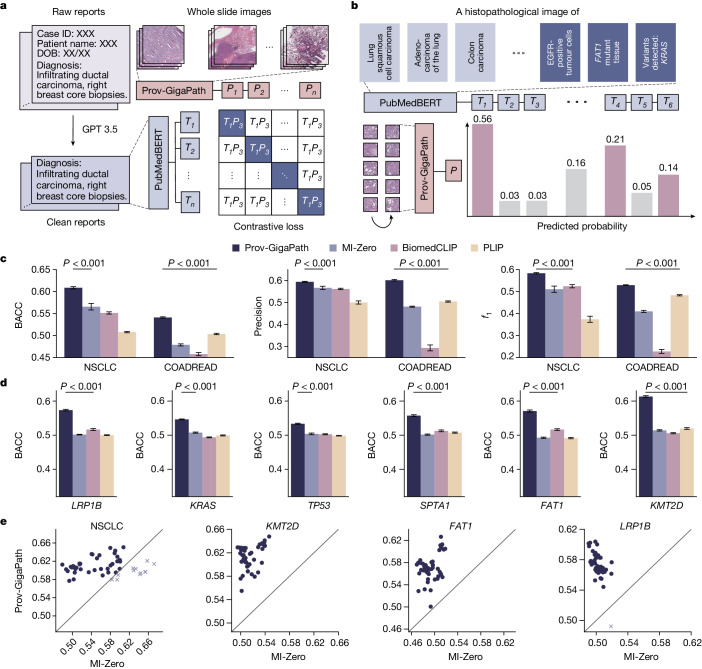
Comparison on image–report alignment. **a**, Flow chart showing the fine-tuning of Prov-GigaPath using pathology reports. Real-world pathology reports are processed using GPT-3.5 from OpenAI to remove information irrelevant to cancer diagnosis. We performed the CLIP-based contrastive learning to align Prov-GigaPath and PubMedBERT. **b**, The fine-tuned Prov-GigaPath can then be used to perform zero-shot cancer subtyping and mutation prediction. The input of Prov-GigaPath is a sequence of tiles segmented from a WSI, and the inputs of the text encoder PubMedBERT are manually designed prompts representing cancer types and mutations. Based on the output of Prov-GigaPath and PubMedBERT, we can calculate the probability of the input WSI being classified into specific cancer subtypes and mutations. **c**, Bar plots comparing zero-shot subtyping performance on NSCLC and COADREAD in terms of BACC, precision and *f*_1_. **d**, Bar plots comparing the performance on mutation prediction using the fine-tuned model for six genes. **c**,**d**, Data are mean ± s.e.m. across *n* = 50 experiments. The listed *P* value indicates the significance for Prov-GigaPath outperforming the best comparison approach, with one-sided Wilcoxon test. **e**, Scatter plots comparing the performance between Prov-GigaPath and MI-Zero in terms of BACC on zero-shot cancer subtyping. Each dot indicates one trial with a particular set of text query formulations.

The promising results of Prov-GigaPath on pathology images further motivated us to explore Prov-GigaPath in multimodal vision–language processing. Prior work on pathology vision–language modelling tends to focus on tile-level alignment of pathology images and text, as their studies were limited by the sources of image–text pairs (textbook examples^7^ or Twitter data^8^). By contrast, we examined slide-level alignment of pathology images and text by leveraging the associated report for each slide (Fig. 4a). Such naturally occurring slide–report pairs can potentially uncover richer slide-level information, but the modelling is considerably more challenging as we do not have fine-grained alignment information between individual image tiles and text snippets. We used the standard cross-modal contrastive loss in continual pretraining of Prov-GigaPath as the visual encoder and PubMedBERT^29^, a state-of-the-art biomedical language model, as the textual encoder (Fig. 4b).

**Extended Data Fig. 9:**
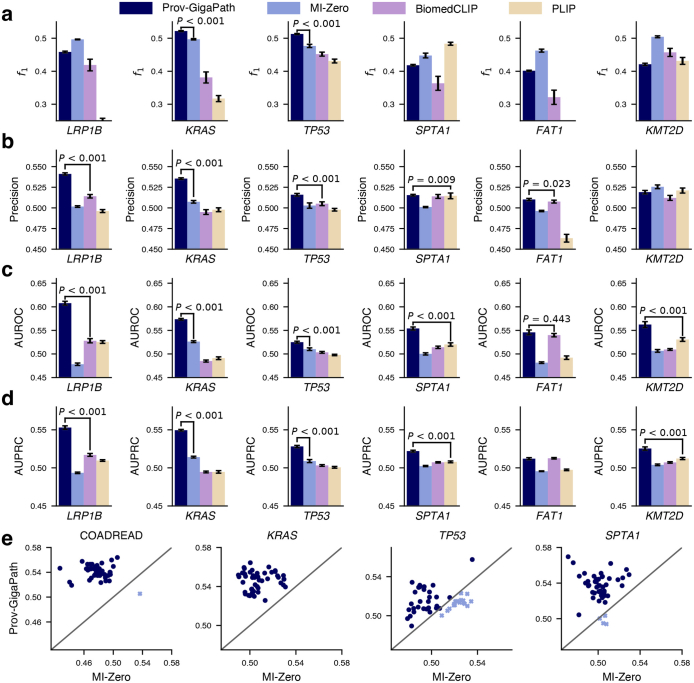
Alignment between pathology reports and images. **a-d**, Bar plots showing the performance of *f*_1_ (**a**), Precision (**b**), AUROC (**c**) and AUPRC (**d**) using fine-tuned *Prov-GigaPath* to predict mutations in the zero-shot learning setting. The error bars show the standard error across n = 50 experiments and the bar centre shows the mean value. The listed *p*-value indicates the significance level that *Prov-GigaPath* outperforms the best comparison approach, with one-sided Wilcoxon test. **e**, Scatter plots comparing *Prov-GigaPath* and MI-Zero on cancer subtyping prediction and mutation prediction in terms of balanced accuracy (BACC).

We evaluated the resulting Prov-GigaPath on zero-shot cancer subtyping in NSCLC and COADREAD following the same setting used in MI-Zero^7^, a state-of-the-art pathology vision–language model. In the zero-shot setting, no training images are provided for any of the target cancer subtypes. Slides and the corresponding cancer subtypes were collected from Prov-Path. Compared with three state-of-the-art pathology vision–language models, Prov-GigaPath attained the best zero-shot classification results on all three metrics in both cancer types (Fig. 4c,e, Extended Data Fig. 9 and Supplementary Fig. 6), suggesting that slide-level alignment enabled by LongNet is indeed advantageous. Prov-GigaPath attained larger improvement on NSCLC than COADREAD, which can be ascribed to the more prevalent presence of lung tissue in Prov-Path. Prov-GigaPath outperformed PLIP by a considerable margin, which potentially reflects the superiority of real-world clinical data over Twitter data.

Next, we examined the possibility of predicting gene mutations using the vision–language pretrained Prov-GigaPath (Fig. 4d,e and Extended Data Fig. 9) in the same zero-shot setting. We adopted the prompts used for cancer subtyping by replacing the cancer type name with the gene name for which we want to predict the binary mutation status. Prov-GigaPath substantially outperformed state-of-the-art pathology vision–language models by a large margin across all six mutations we have examined (*P* value < 0.001).(Fig. 4d,e). The improvement of our approach is larger on mutation prediction than on cancer subtyping, which may be partially attributable to richer mutation information in pathology reports from real-world data compared with text commentary in Twitter^8^ and scientific papers^50^. To our knowledge, this is the first time zero-shot gene mutation prediction was evaluated on pathology vision–language modelling. The promising performance of Prov-GigaPath on this novel task bodes well for potential future applications in studying rare cancer types and new mutations.

## Discussion

We have introduced Prov-GigaPath, a pathology foundation model for a broad range of digital pathology applications. Prov-GigaPath was pretrained on a large real-world dataset Prov-Path derived from Providence health system with diverse types and qualities. Prov-Path is substantially larger than TCGA, comprising 1,384,860,229 image tiles from 171,189 whole pathology slides of around 30,000 patients. We proposed GigaPath for pretraining, which adapted the cutting-edge LongNet^5^ as the vision transformer to facilitate ultra-large-context modelling of gigapixel WSIs. In comprehensive evaluation on both Providence and TCGA datasets, we demonstrated state-of-the-art performance for Prov-GigaPath on a variety of pathomics and cancer subtyping tasks, as well as on vision–language processing. Prov-GigaPath has the potential to assist clinical diagnostics and decision support, and GigaPath can potentially be applicable to broader biomedical domains for efficient self-supervised learning from high-resolution images.

We noted substantial variability in the performance of our method across different tasks. First, the performance on subtyping is substantially better than the performance on mutation prediction. Although different tasks are not comparable owing to the number of training samples, our observations suggest that image-based mutation prediction is more challenging. One particular reason could be that the pathology image information is not enough to predict certain mutations. Therefore, we plan to utilize other modalities and features to enhance the prediction in the future. Nevertheless, our method outperforms existing approaches on mutation prediction tasks, offering an opportunity to improve diagnostics and prognostics. Moreover, we found that foundation models, including our method and competing approaches, are much more effective than task-specific models (for example, SL-ImageNet in Supplementary Fig. 4), necessitating the self-supervised learning framework in these foundation models. We currently select a magnification of 20 during preprocessing. A larger magnification will quadruple the processing time but also reveal more details of the image. Therefore, we are interested in exploring other magnifications in the future. Scaling laws have been observed in large language models when modelling text data. We have observed that GigaPath pretrained on the larger Prov-Path data outperforms GigaPath pretrained on the smaller TCGA data (Extended Data Fig. 6). Despite having different model architectures, we have also observed that GigaPath, which has more parameters, outperforms HIPT when both are pretrained on Prov-Path. These two results indicate the effectiveness of larger pretraining data and larger models, which partly indicate that the model performance may further improve with more pretraining tokens. We are interested in further validating scaling laws in the context of pathology foundation models by comparing models at different sizes and pretraining data at different sizes.

Although initial results are promising, growth opportunities abound. First, it would be interesting to study scaling laws^51^ on the pathology foundation models by comparing the performance using different sizes of vision transformers. In particular, we found that a smaller version of Prov-GigaPath using 23 million parameters also attained superior performance than existing approaches, demonstrating the application of two models in real-world clinics: a small model for fast inference and fine-tuning, and a large model (Prov-GigaPath) for more accurate inference. Second, the pretraining process can be further optimized. In slide-level self-supervised learning, we froze the tile-level encoder when pretraining the slide-level encoder to reduce memory cost, which may be suboptimal. We plan to explore end-to-end pretraining with larger graphics processing unit (GPU) clusters, on which we can compute image encoding on the fly and fine-tune all the way. Third, we conducted an initial exploration on vision–language pretraining and demonstrated promising results in zero-shot subtyping and mutation prediction, but this remains far away from the potential to serve as a conversational assistant for clinicians. In future, we plan to incorporate advanced multimodal learning frameworks, such as LLaVA-Med^52^, into our work.

## Methods

### Preprocessing WSIs

We first established our preprocessing pipeline for the 171,189 H&E-stained^53^ and immunohistochemistry^54^ pathology slides. The statistics of slides and patients for each organ are shown in Supplementary Figs. 1 and 2. First, we performed tissue segmentation to filter background regions. Following HIPT, we ran the Otsu^55^ image thresholding at a downsampled resolution (for example, 1,024 pixels) for its computational efficiency and effectiveness in differentiating tissues from the background. Second, we resized the WSIs to a standard resolution of 0.5 μm per pixel (MPP)—that is, 20× magnification using the pyvips library. This step is necessary because some slides have higher resolution depending on the scanner settings. Finally, the images were cropped into 256 × 256-pixel tile images. Tiles with an occupancy value of less than 0.1, determined by the Otsu algorithm, were discarded to focus on tissue-covered regions. We performed these operations on a cluster of up to 200 nodes, where each node was equipped with 32 CPU cores and 256 GB RAM, completing preprocessing in about 157 hours. Tasks were parallelized, so that each node processed a set of tiles independently. Finally, we collected 1,384,860,229 tiles in total, with the number of tiles in each WSI shown in Supplementary Fig. 3.

### Details of Prov-GigaPath pretraining

Prov-GigaPath tile encoder used the ViT model architecture with standard DINOv2 settings^24^. We pretrained the model on 1,384,860,229 segmented tiles, treating each tile as one data instance. The base learning rate in DINOv2 pretraining was set to 4 × 10^−3^. We set the batch size on each GPU device to 12, with a total effective batch size of 384. Prov-GigaPath slide encoder used the LongNet model architecture with standard settings^5^. For discretizing the tile coordinates, we set the grid size *d*_grid_ to 256 and the number of rows and columns to *n*_grid_ to 1,000. For the input sequence augmentations, we set the cropping ratio to 0.875. The moving distances were randomly generated with a uniform distribution by keeping all tiles within the created grid overlay. We horizontally flipped the tile coordinates for each slide with a 0.5 probability. To pretrain our Prov-GigaPath slide encoder with the masked autoencoder, we set the learning rate to 5 × 10^−4^ and the batch size on each GPU device to 4. We also set the training epochs to 30 with the initial epoch as the warmup phase. The slide encoder pretraining utilized 16 nodes with 4 × 80 GB A100 GPUs and was completed in approximately 2 days (3,072 A100 GPU hours). The inference duration for a WSI is on average 0.7 s, including 0.4 s on computing tile embeddings and 0.3 s on LongNet inference.

### Competing methods and benchmarks

We compared Prov-GigaPath to 4 comparison approaches. HIPT^35^ was a released model pretrained on 10,678 gigapixel WSIs from TCGA. It utilized a hierarchical image pyramid transformer architecture with 256 × 256 and 4,096 × 4,096 image views. We can also view the HIPT model as a tile encoder with an additional embedding aggregation encoder on the 4,096 × 4,096 view. Since it used the DINO self-supervised learning approach to train the 256 × 256 image encoder and 4,096 × 4,096 image encoder, the tile encoder pretraining of HIPT was the same as Prov-GigaPath. The key difference between HIPT and Prov-GigaPath was the aggregation mechanism. Prov-GigaPath approached aggregation using long-sequence representation learning with a slide encoder, whereas HIPT employed a second-stage ViT on the 4,096 × 4,096 image view. CtransPath^41^ combined a CNN model with a multi-scale SwinTransformer. CtransPath used a semantically relevant contrastive-learning objective to pretrain the model, which treated each input image and its augmentation views as positive pairs and ${\mathcal{S}}$ retrieved semantically relevant images as pseudo-positive pairs. REMEDIS^42^ used a Resnet as the backbone and pretrained with the SimCLR approach on 50 million pathology images randomly sampled from 29,018 TCGA slides. In our experiments, we selected the Resnet 152 × 2 model for evaluation.

We fine-tuned Prov-GigaPath and other baseline models on diverse downstream tasks. For Prov-GigaPath, we froze the tile encoder and only fine-tuned the LongNet slide-level encoder. For each slide, LongNet produces a set of contextualized tile embeddings. These are aggregated using a shallow ABMIL layer to obtain the slide embeddings, which are then used in additional classifiers for downstream prediction tasks. When applying the HIPT model, we followed the default setting by freezing both the 256 × 256 and 4,096 × 4,096 image encoder and tuning the parameters of the additional transformer layer and ABMIL layer. Since both CtransPath and REMEDIS are tile-level encoders, we directly applied one ABMIL layer to get slide-level embeddings and mainly tuned the ABMIL layer and classifier.

### Mutation prediction

From Prov-Path, we constructed 5-gene mutation prediction tasks: pan-cancer 18 biomarkers prediction, LUAD 5-gene mutation prediction, pan-cancer 5-gene mutation prediction, LUAD 5-gene mutation prediction on TCGA and overall TMB prediction (Supplementary Tables 7 and 9). The 18 biomarkers prediction is an 18-class multi-label classification problem, with each class being either a mutation or PD-L1. The positive status for each gene indicates that it is mutated or that PD-L1 (encoded by *CD274*) is highly expressed. The 5-gene mutation prediction tasks are 5-class classification problems. The 5-gene mutation prediction tasks including 5 genes (*EGFR*, *FAT1*, *KRAS*, *TP53* and *LRP1B*) are formulated as a multi-label prediction task where the model was asked to predict mutation status for all genes. The overall TMB prediction is a 2-class classification (High TMB versus Low TMB). We formulated this task as an image binary classification task where each image is annotated as ‘High TMB’ and ‘Low TMB’ based on the number of somatic mutations of the tumour^56^. Such evaluations reflect the the capability of the model to extracting diverse molecular patterns on the WSIs. For each patient, who typically has multiple WSIs, we selected the largest WSI. This naturally enabled patient-level stratification when splitting the datasets into training, validation, and test sets. We fine-tuned Prov-GigaPath model with the base learning rate of 2 × 10^−3^ and the weight decay of 0.01. Following the default settings in HIPT, we trained the comparison models with a learning rate of 2 × 10^−4^. The training batch size for all approaches was set to 1 with 32 gradient accumulation steps. We trained all approaches for 20 epochs. The performances were evaluated in terms of the AUROC and AUPRC using the 10-fold cross-validation.

### Cancer subtyping

We conducted the subtyping evaluations on nine cancer types, including NSCLC (LUAD versus LUSC), BRCA (IDC versus ILC), RCC (CCRCC versus PRCC versus CHRCC), COADREAD (COAD versus READ), HB (CHOL versus HCC), DIFG (GBM versus ODG versus AODG versus HGGNOS versus AASTR), OVT (CCOV versus EOV versus HGSOC versus LGSOC versus MOV versus OCS), CNS (ATM versus MNG) and EGC (ESCA versus GEJ versus STAD); details and definitions are provided in Supplementary Tables 8 and 9. We fine-tuned the Prov-GigaPath with the base learning rate of 4 × 10^−3^, the weight decay of 0.001, and the layer-wise learning rate decay of 0.9. The training hyperparameters were chosen based on performance on the validation set. All models were fine-tuned for 20 epochs and evaluated using the tenfold cross-validation. For the Prov-GigaPath, we additionally added a shortcut to the slide-level encoder to pay more attention to tile-level features.

### Vision–language alignment

We constructed 17,383 pathology WSI-reports pairs and employed the OpenCLIP codebase for vision–language processing. Since real-world pathology reports are noisy and lengthy, we first clean the raw pathology reports by removing information irrelevant to cancer diagnosis, including hospital location, doctor name, and patient name. Specifically, we first clustered the clinical reports into four clusters using *k*-means and picked the cluster centres as four representative reports. We then manually cleaned these four reports and obtained four pairs of original and cleaned reports. We used these four reports as in-context learning examples and asked GPT-3.5 to clean all other reports according to these four in-context learning examples (Supplementary Fig. 9). The distributions of the overall token length before and after the filtering are shown in Supplementary Fig. 10. The text embeddings were calculated using the text-embedding-ada-002 model from OpenAI. Finally, we constructed 17,383 vision–language pairs of WSI and the cleaned reports. We hold out 20% of the patients from CLIP pretraining for zero-shot prediction tasks. We set the learning rate of the CLIP training to 5 × 10^−4^ and the batch size to 32. We trained both the visual encoder and the text encoder for 10 epochs with the first 100 iterations as the warmup stage.

In zero-shot prediction tasks, we chose the MI-Zero (PubMedBERT)^7^, BiomedCLIP^50^ and PLIP^8^ as the comparison models. MI-Zero (PubMedBERT) was trained on 33,480 pathology image-caption pairs curated from educational resources and ARCH dataset. It is a multiple instance learning-based zero-shot transferring approach by aggregating multiple tiles with a top *K* pooling strategy. BiomedCLIP was trained on 15 million biomedical domain-specific image-caption pairs from research articles. PLIP was a pathology domain-specific vision–language pretrained model using image–text pairs from Twitter. We evaluated the comparison approaches and Prov-GigaPath on NSCLC and COADREAD subtyping tasks and *LRP1B*, *KRAS*, *TP53*, *SPTA1*, *FAT1* and *KMT2D* mutation status prediction. We followed the settings and prompt templates in MI-Zero^7^ and evaluated these approaches with 50 randomly sampled prompts set.

### Reporting summary

Further information on research design is available in the Nature Portfolio Reporting Summary linked to this article.

## Online content

Any methods, additional references, Nature Portfolio reporting summaries, source data, extended data, supplementary information, acknowledgements, peer review information; details of author contributions and competing interests; and statements of data and code availability are available at 10.1038/s41586-024-07441-w.

### Supplementary information

Supplementary Information The Supplementary Information text provides descriptions of the overview of Prov-GigaPath and the LongNet-based two-stage pretraining. Supplementary Figs 1–3 provide the statistics of tiles, slides and patients in Prov-Path. Supplementary Fig. 4 shows the comparison between different tile-level pretraining methods. Supplementary Fig. 5 shows the ablation studies of Prov-GigaPath on cancer subtyping tasks. Supplementary Fig. 6 shows the AUROC and AUPRC of zero-shot cancer subtyping tasks. Supplementary Fig. 7 presents a comparison between the LongNet and Vanilla Attention with FlashAttention. Supplementary Fig. 8 gives one illustration of dilated attention. Supplementary Fig. 9 shows the prompt template we used when processing and denoising the real-world pathology reports using OpenAI’s GPT-3.5. Supplementary Fig. 10 shows the distributions of token length before and after preprocessing. Supplementary Table 1 shows the overview of existing pathology image foundation models and Prov-GigaPath. The Supplementary Table 2 compares Prov-GigaPath with state-of-the-art pathology foundation models on 25 tasks in pathomics and cancer subtyping using AUROC. Supplementary Tables 3-5 show the distribution of gender, age and race of patients in Prov-Path. Supplementary Table 6 shows the mutation rates of patients. Supplementary Tables 7-8 show the number of slides for each class in each gene mutation prediction task and cancer subtyping task. Supplementary Table 9 provides the number of patients in the training, validation and test set across different tasks in Prov-Path.

Reporting Summary

## Data Availability

The pathology imaging data used for the pretraining were created from oncology pathology slides at Providence. The associated clinical data used for fine-tuning and testing were obtained from the corresponding medical records. These proprietary data cannot be made publicly available. Researchers may obtain a de-identified test subset from Providence Health System by reasonable request and subject to local and national ethical approvals. To help researchers use our model, we provide a de-identified subset of our data at 10.5281/zenodo.10909616 (ref. ^57^) and 10.5281/zenodo.10909922 (ref. ^58^) for a few patients. We also collected publicly available TCGA WSIs from the NIH Genomic Data Commons Data Portal. The TCGA-LUAD dataset, comprising whole pathology slides and labels, is available via the NIH Genomic Data Commons portal at https://portal.gdc.cancer.gov/projects/TCGA-LUAD.

## Code availability

Prov-GigaPath is a vision transformer model created by tile-level pretraining using DINOv2, followed by slide-level pretraining using masked autoencoder and LongNet, on more than 170,000 whole slides with more than a billion pathology image tiles. The pathology slides were stripped of the identification barcodes before pretraining. Prov-GigaPath can be accessed at https://github.com/prov-gigapath/prov-gigapath, including the model weights and relevant source code. We include detailed methods and implementation steps in the Methods and Supplementary Information to enable independent replication.
