# Supplementary material for: A whole-slide foundation model for digital pathology from real-world data
Source: Nature. 2024 May 22;630(8015):181–8. doi: 10.1038/s41586-024-07441-w (PMC11153137; doi:10.1038/s41586-024-07441-w)
Supplement: Supplementary file 1 — The Supplementary Information text provides descriptions of the overview of Prov-GigaPath and the LongNet-based two-stage pretraining. Supplementary Figs 1–3 provide the statistics of tiles, slides and patients in Prov-Path. Supplementary Fig. 4 shows the comparison between different tile-level pretraining methods. Supplementary Fig. 5 shows the ablation studies of Prov-GigaPath on cancer subtyping tasks. Supplementary Fig. 6 shows the AUROC and AUPRC of zero-shot cancer subtyping tasks. Supplementary Fig. 7 presents a comparison between the LongNet and Vanilla Attention with FlashAttention. Supplementary Fig. 8 gives one illustration of dilated attention. Supplementary Fig. 9 shows the prompt template we used when processing and denoising the real-world pathology reports using OpenAI’s GPT-3.5. Supplementary Fig. 10 shows the distributions of token length before and after preprocessing. Supplementary Table 1 shows the overview of existing pathology image foundation models and Prov-GigaPath. The Supplementary Table 2 compares Prov-GigaPath with state-of-the-art pathology foundation models on 25 tasks in pathomics and cancer subtyping using AUROC. Supplementary Tables 3-5 show the distribution of gender, age and race of patients in Prov-Path. Supplementary Table 6 shows the mutation rates of patients. Supplementary Tables 7-8 show the number of slides for each class in each gene mutation prediction task and cancer subtyping task. Supplementary Table 9 provides the number of patients in the training, validation and test set across different tasks in Prov-Path. [file 41586_2024_7441_MOESM1_ESM.pdf]

nature portfolio

https://doi.org/10.1038/s41586-024-07441-w

Supplementary information

A whole-slide foundation model for digital pathology from real-world data

In the format provided by the authors and unedited

Supplementary Information

Overview of ***Prov-GigaPath***

The goal of the pathology foundation model is to learn an encoder from a large number of pathology images so that this encoder can be quickly adapted to diverse downstream tasks when no labeled data (zero-shot) or a small amount of labeled data (few-shot) are provided for these tasks. To learn this encoder, we use a sequence-based self-supervised embedding framework. In particular, we segment the whole-slide image $\mathbf{I}$ into a sequence of tiles $\{\mathbf{I}_l\}_{l=1}^{L} \in \mathbb{R}^{L\times 3\times 256\times 256}$, where 3 and 256 represent the image channel number and image size respectively. Tiles without substantial tissue occupancy are excluded. Using the terminology from natural language modeling, we treat each small tile as a token and employ a tile encoder $\mathbf{E}_{\mathrm{T}}$ to calculate tile embeddings $\mathbf{z}_l = \mathbf{E}_{\mathrm{T}}(\mathbf{I}_l) \in \mathbb{R}^{d_{\mathrm{T}}}$. The tile encoder, typically a ViT model[1], individually embeds each tile on the WSI. Similar to the sentence encoder in language modeling, we further employ a slide encoder to generate contextualized tile embeddings. We denote the slide encoder as $\mathbf{E}_{\mathrm{S}}$ and the contextualized tile embeddings from $\mathbf{E}_{\mathrm{S}}$ as $\{\hat{\mathbf{z}}_l\}_{l=1}^{L}$. In a self-supervised setting, our task is to learn a tile encoder $\mathbf{E}_{\mathrm{T}}$ and a slide encoder $\mathbf{E}_{\mathrm{S}}$ to produce $\{\hat{\mathbf{z}}_l\}_{l=1}^{L}$, which can be quickly adapted to diverse downstream tasks by only fine-tuning the slide encoder. The contextualized WSI tile embeddings are then defined as:

$$\{\hat{\mathbf{z}}_l\}_{l=1}^{L} = \mathbf{E}_{\mathrm{S}}(\{\mathbf{E}_{\mathrm{T}}(\mathbf{I}_l)\}_{l=1}^{L}). \tag{1}$$

Let's define the set of k downstream tasks as $\mathcal{T} = \{\tau\}^k$. Based on learned contextualized embeddings, each task τ is to fit a predictive model f_τ on the downstream dataset $\{(\mathbf{I}, h_\tau)\}^n$:

$$\hat{h} = f_\tau(\mathbf{E}_{\mathrm{S}}(\{\mathbf{E}_{\mathrm{T}}(\mathbf{I}_l)\}_{l=1}^{L})), \tag{2}$$

where h_τ and $\hat{h}$ are the ground truth and predicted labels from task τ. For example, h_τ represents whether a gene is mutated in mutation prediction. In cancer subtyping, h_τ repsents a specific cancer subtype.

LongNet-based two-stage pretraining

GigaPath uses a novel two-stage pretraining approach. At the first stage, it pretrains the tile encoder on 256×256 size tiles. We employ the recent advancement in self-supervised learning, DINOv2 [2], to train the tile encoder $\mathbf{E}_{\mathrm{T}}$ in *Prov-GigaPath* on all individual tiles. DINOv2 adopts a discriminative self-supervised learning objective, which can be viewed as the combination of the image-level objective, patch-level objective and the KoLeo regularizer [2]. DINOv2 incorporates a student-teacher architecture and multi-crop augmentation to learn high-quality image representations. In our implementation, it crops the same 256×256 image into a set of 96×96 local views and 224×224 global views. By providing the student model with all crops and the teacher with only global views, the image-level objective encourages the model learns ”local-to-global” correspondences [3]. DINOv2 also masks patches on the images given to the student model, while passing the complete images to the teacher. The patch-level objective is to minimize the discrepancy between the features of masked patches from the student and teacher model [2]. The patch-level objective is also known as the iBOT loss [4]. To make sure the uniform span of learned image representations in the same batch, the KoLeo regularizer maximizes the minimum distance bewteen the representation vectors within one batch [2]. To better avoid the model collapse, DINOv2 advances the teacher softmax centering to a Sinkhorn-Knopp centering normalization [5]. We followed the standard DINOv2 settings in all the experiments. We also explored alternative settings and found that a smaller model with longer pretraining may yield comparable results while incurring substantially lower cost at inference.

In the second stage, we pretrain a slide encoder to contextualize each tile on the WSI. To efficiently calculate the contextualized tile embeddings, we opt for the recently advanced LongNet model [6] as our slide encoder $\mathbf{E}_{\mathrm{S}}$. This model is a subvariant of the transformer architecture and is notable for its dilated attention mechanism, which demonstrated proficiency in handling up to 1 billion tokens with multiple GPU devices [6]. We found that LongNet is faster than FlashAttention [7] (**Supplementary Fig. 7**), allowing us to accelerate the training procedure.

The core idea of dilated attention is to segment the long sequence and sparsify each segment, which can effectively reduce the huge memory cost when processing large whole slide images. In the dilated attention, it

first segments the input sequence with a length L into $\frac{L}{w}$ segments with equal length w. It then sparsifies each segment with an interval r along the sequence length (**Supplementary Fig. 8**). The attention operation is first performed on the sparsifed segments and then scattered to the original segment length. Finally, it concatenates attention outputs across all segments as the output of dilated attention. This dilated attention operation can reduce the memory cost by $\frac{N}{w}r^2$[6], supporting us in modeling tens of thousands of tiles per GPU device. Intuitively, setting (w, r) can lead to different attention patterns. For example, when we set a small segment length and the interval to 1, the dilated attention could accurately capture local patterns within each segment. When we set a large segment length with large interval, the model can approximate interactions between distant regions on the whole slide image, while maintaining manageable memory costs. Based on this idea, we optimizes to chose a set of (w, r) pairs, including $\{(512, 1), (1024, 2), (2048, 4)\}$. To combine the attention outputs from these three (w, r) pairs, the LongNet model calculates the weighted average of the three outputs, where the weights are the denominator of the softmax operation in each dilated attention.

The slide encoder takes each tile image embedding $\mathbf{z}_l$ from the tile encoder as the input token. Additionally, we also add position embeddings for each tile embedding with its coordinate (x_l, y_l) on the WSI. To discretize the continuous tile coordinates, we overlay a d_{grid}-sized grid on the image, defined by n_{grid} rows and columns. Then we map the continuous tile coordinates to grid vertices using:

$$(\widehat{x}_l, \widehat{y}_l) = (\lfloor \frac{\min(x_l, d_{\text{grid}} \times n_{\text{grid}})}{d_{\text{grid}}} \rfloor, \lfloor \frac{\min(y_l, d_{\text{grid}} \times n_{\text{grid}})}{d_{\text{grid}}} \rfloor). \quad (3)$$

Then we follow the standard ViT architecture to transform the mapped grid vertices into the 2D positional embeddings: Even though we have transformed each tile image into a compact embedding followed by a memory efficient LongNet, the slide-level pretraining of $\mathbf{E}_\text{S}$ still results in a high memory cost. Here we choose the reconstruction-based masked autoencoder approach to pretrain $\mathbf{E}_\text{S}$, which can work well with a small batch size. Moreover, employing a high masking ratio of 75% in the masked autoencoder can also reduce the encoder memory cost by removing masked tokens from input to the encoder, enabling more rapid iteration over all 171,189 WSIs. Compared to the masked autoencoder [8] which uses the pixel values of masked tiles as the reconstruction target, our LongNet model built upon the tile embeddings optimizes the cosine similarity between predictions and the original embeddings on masked tokens. For each input tile embedding sequence, we design several augmentation transformations. To implement the cropping operations, we reorder the tile images by ascending x-coordinate, followed by the y-coordinate, and then select a continuous chunk on the reordered sequence. The chunk size is calculated from the cropping ratio. We also add random biases to the tile coordinates to mimic the moving transformation and flip the coordinates to implement the horizontal flipping. Finally, we add Gaussian noise to the tile embeddings to improve the robustness of our approach.

In our implementation, we used the DINOv2 official codebase (https://github.com/facebookresearch/dinov2/) to pretrain our tile encoder. We used the LongNet implementation in torchscale (https://github.com/microsoft/torchscale). We used OpenCLIP (https://github.com/mlfoundations/open_clip) to train the vision-language alignment model. Other packages used in our codebase include torch 2.0.0, torchvision 0.15.0, tensorboard 2.15.1, timm 0.9.12, xformers 0.0.18, einops 0.7.0, fairscale 0.4.13, huggingface-hub 0.19.4. When evaluating our model, we used scikit-learn 1.3.2, scipy 1.11.4 and numpy 1.24.1. We used matplotlib 3.3.0 to visualize the data. We released both our model weights and codebase in the github repo (See **Code availability**). Our codebase supports running inference of the model to produce slide embeddings. It can also be evaluated on the mutation benchmarks. We also release a demo script to guide users to run our pipeline.

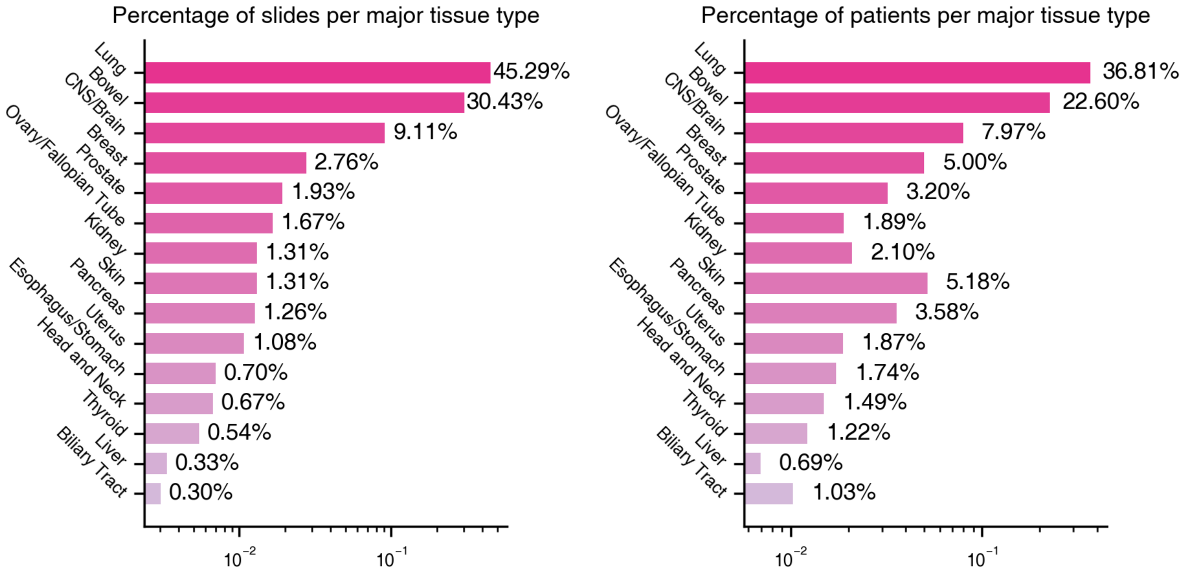

Supplementary Figure 1: **The statistics of slides and patients in *Prov-Path*.** Bar plots showing the percentage of slides and the percentage of patients for each organ. 15 organs with the most patients are shown here.

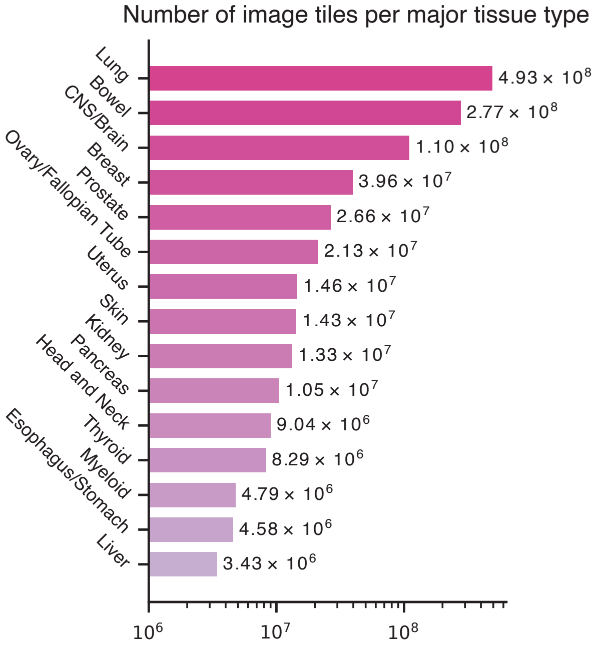

Supplementary Figure 2: **Number of tiles in *Prov-Path*.** Bar plot showing the number of tiles for each organ. 15 organs with the most patients are shown here.

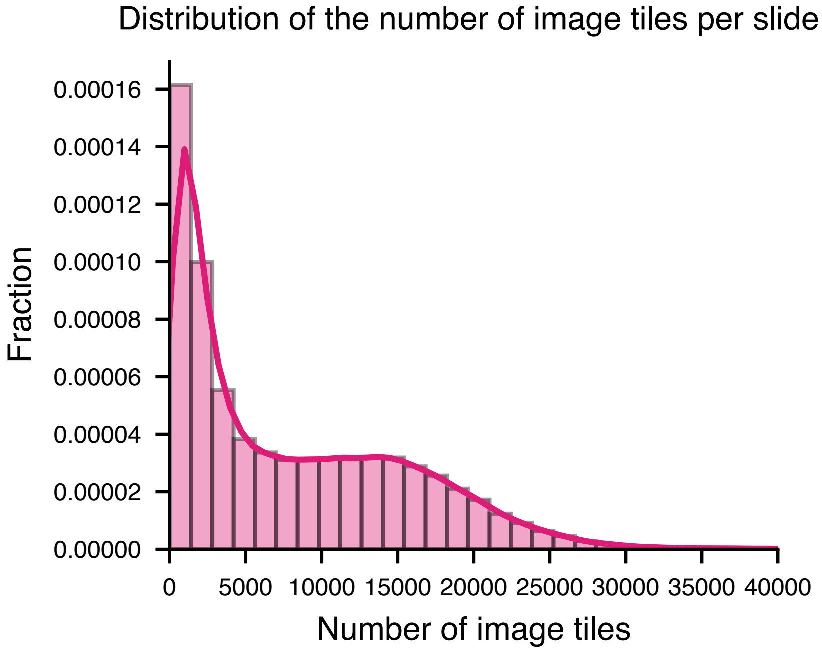

Supplementary Figure 3: **Distribution of the number of image tiles per slide.** Bar plot showing the distribution of the number of tiles in each slide.

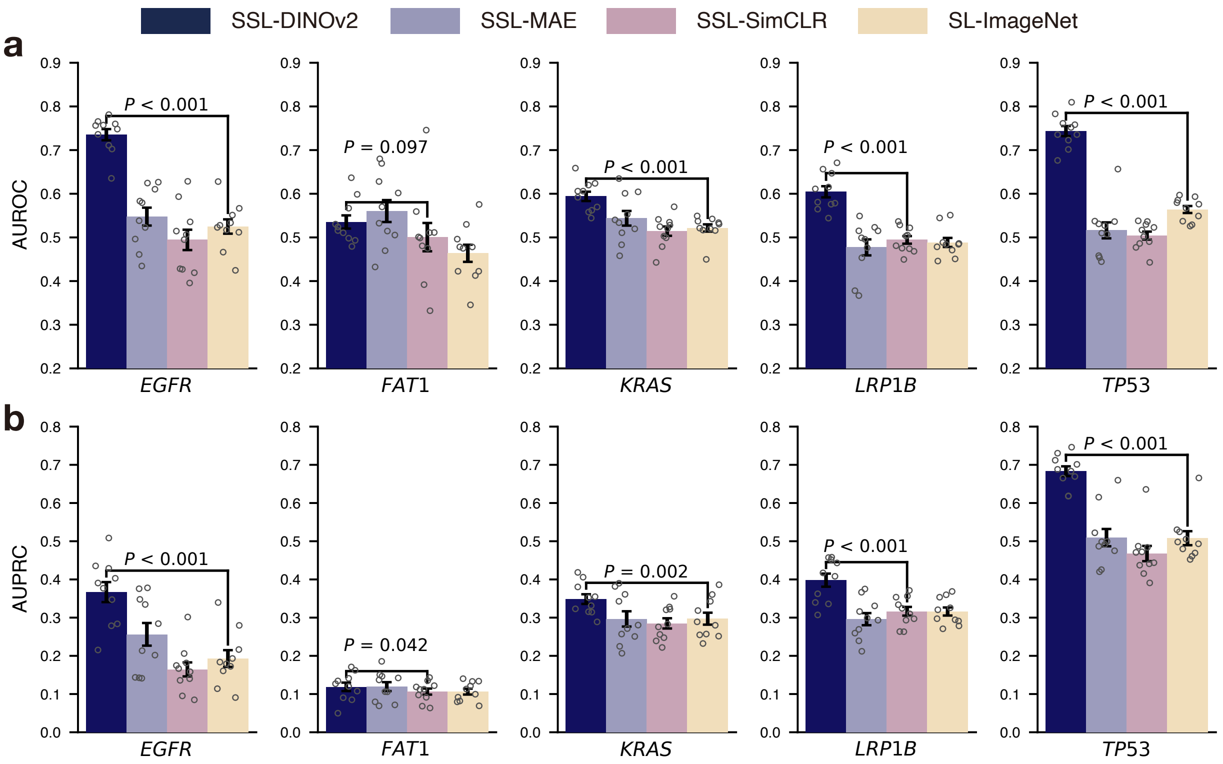

Supplementary Figure 4: **Comparison of different tile-level pretraining methods. a,b**, Bar plots showing the AUROC (**a**) and AUPRC (**b**) on LUAD 5-gene mutation prediction in TCGA using different tile-level pretraining methods, including DINOv2 used by *GigaPath* (SSL-DINOv2), masked autoencoders (SSL-MAE), SimCLR (SSL-SimCLR) and task-specific supervised fine-tuning initialized using an ImageNet-trained model (SL-ImageNet). SSL denotes self-supervised learning. SL denotes supervised learning. The error bars show the standard error across n=10 independent experiments and the bar centre shows the mean value. The listed p-value indicates the significance level that SSL-DINOv2 outperforms the best comparison approach, with one-sided Wilcoxon test.

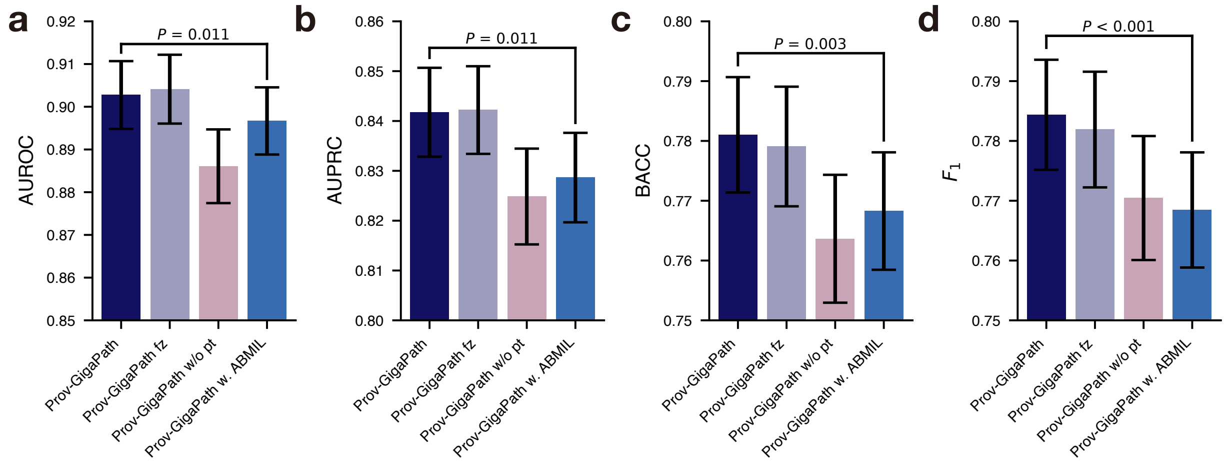

Supplementary Figure 5: **Ablation studies of *Prov-GigaPath*.** Bar plots showing test performance for cancer subtyping under AUROC (**a**), AUPRC (**b**), balanced accuracy (**c**) and $F1$ (**d**). Four variants of *GigaPath* are compared: original (*Prov-GigaPath*), frozen LongNet (*Prov-GigaPath* fz), without slide-level pretraining (*Prov-GigaPath* w/o pt), replacing LongNet with ABMIL (*Prov-GigaPath* w. ABMIL). We conduct the significance test between the original *Prov-GigaPath* and *Prov-GigaPath* w. ABMIL. The error bars show the standard error across n=10 independent experiments and the bar centre shows the mean value. The listed p-value indicates the significance level that *Prov-GigaPath* outperforms *Prov-GigaPath* w. ABMIL, with one-sided Wilcoxon test.

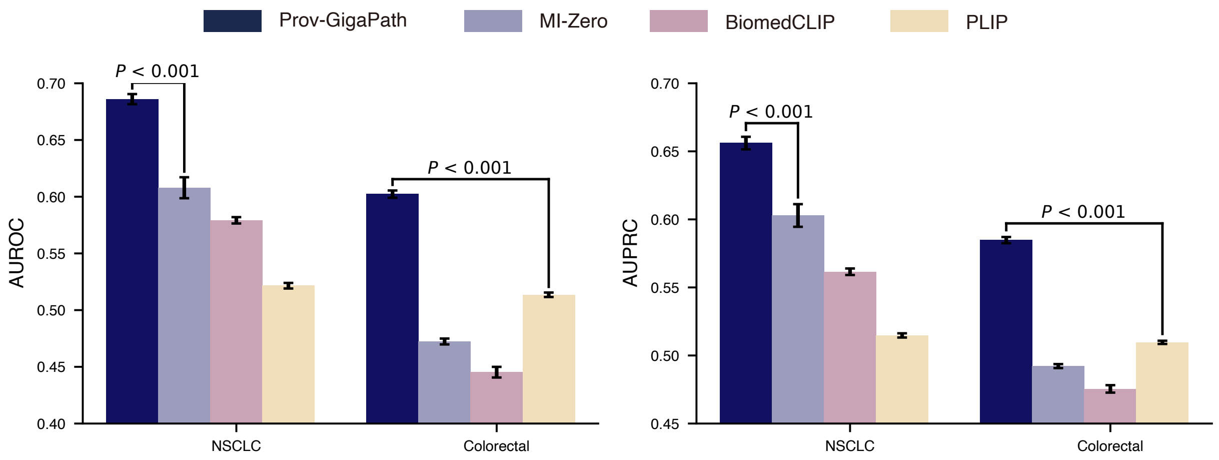

Supplementary Figure 6: **Zero-shot cancer subtyping based on vision-language pretraining using image-report pairs.** Bar plots showing the performance of cancer subtyping on NSCLC and COAD-READ in the zero-shot learning setting. The error bars show the standard error across 50 experiments and the bar centre shows the mean value. The listed p-value indicates the significance level that *Prov-GigaPath* outperforms the best comparison approach, with one-sided Wilcoxon test.

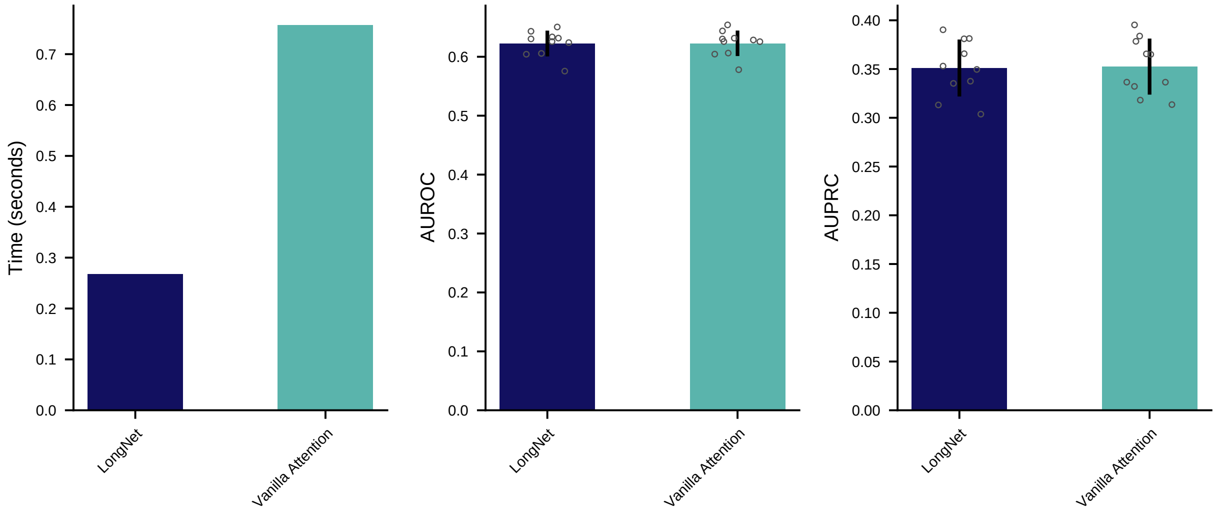

Supplementary Figure 7: **Comparison between LongNet and Vanilla Attention with FlashAttention.** Bar plots comparing the speed and the performance between LongNet and Vanilla Attention. Both LongNet and Vanilla Attention used FlashAttention. The time is average per slide in one iteration. The performance is evaluated on LUAD 5-gene mutation prediction in Providence. LongNet achieves faster computational time while obtaining comparable performance with Vanilla Attention with FlashAttention. The error bars show the standard error across 10 experiments and the bar centre shows the mean value.

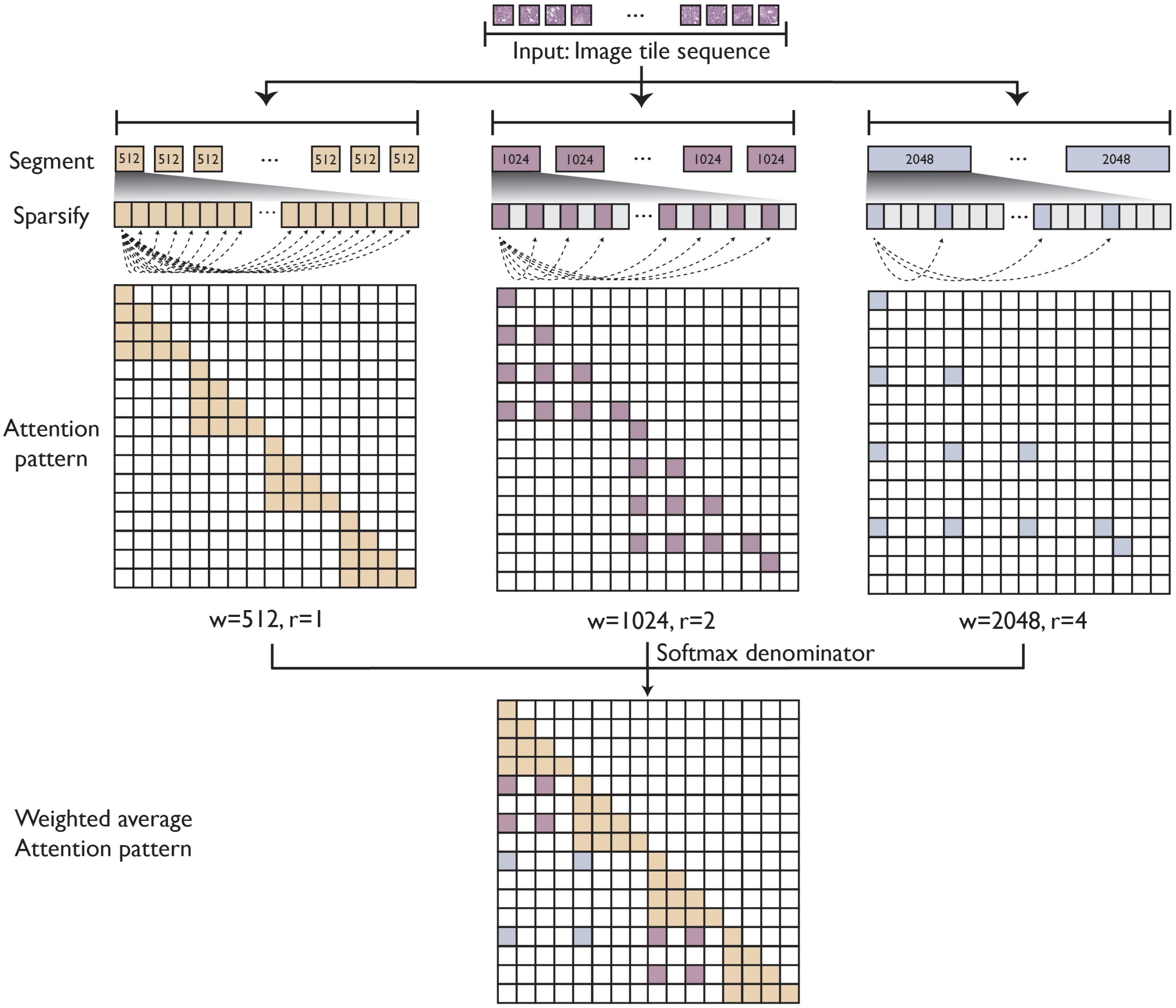

Supplementary Figure 8: **Illustration of dilated attention.** The dilated attention first separates each input tile sequence into segments, with the segment length w. Within each segment, it sparsifies the attention calculation with an interval r. We used a set of three (w, r) pairs, including $(512, 1)$, $(1024, 2)$, and $(2048, 4)$ to extract the local and global interactions on the whole slide images. The LongNet model uses the weighted average of attention outputs from different (w, r) pairs as the output, with the weights as the denominator of the attention softmax for each (w, r).

Prompt for preprocessing real-world pathology report

You're a helpful AI assistant. Help me clean pathology reports for cancer analysis. Please remove information that are not relevant to cancer diagnosis. Here are a few examples:
Input: {pathology report example 1}
Output: {manually cleaned pathology report example 1}
Input: {pathology report example 2}
Output: {manually cleaned pathology report example 2}
Input: {pathology report example 3}
Output: {manually cleaned pathology report example 3}
Input: {pathology report example 4}
Output: {manually cleaned pathology report example 4}
Input: {pathology report}
Output:

Supplementary Figure 9: **The prompt template for processing and denoising real-world pathology reports using OpenAI's GPT-3.5.** We first selected four representative raw reports and manually cleaned them. The raw reports and cleaned reports were provided as in-context learning examples in the above template for GPT-3.5 to clean other reports.

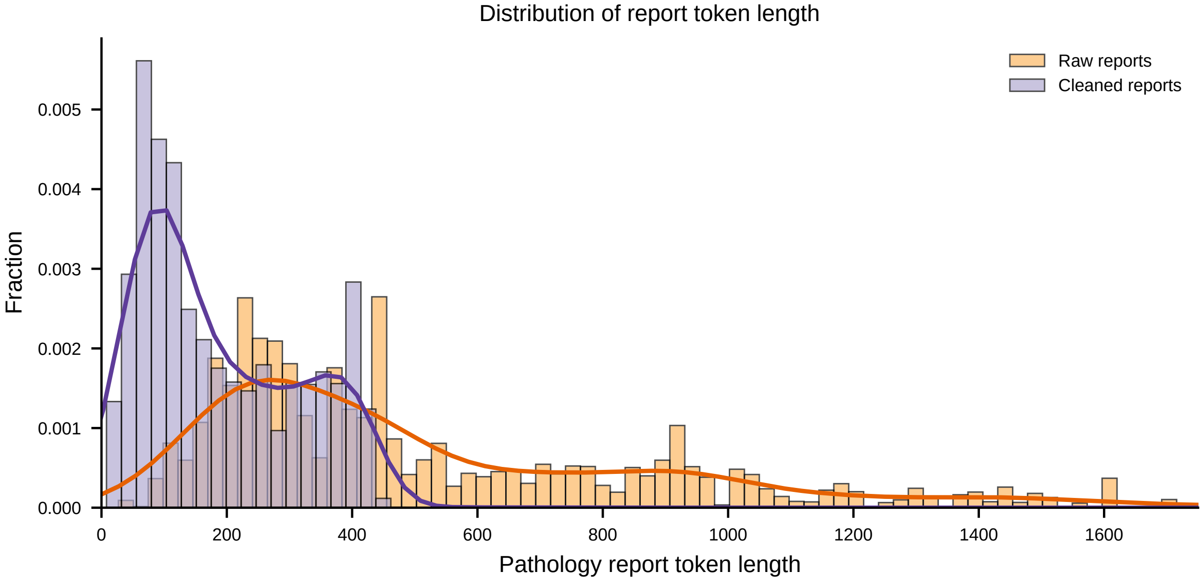

Supplementary Figure 10: **Distributions of token length before and after pre-processing.** Bar plot showing the distributions of the number of tokens in the report before and after the cleaning using GPT-3.5.

Model	Model Architecture	Data Size (#tiles)	Pretraining Input Size	Data Source
HIPT [1]	ViT (DINOv1)	104 million	Region-level ($4,096 \times 4,096$)	TCGA
CtransPath [9]	CNN/Swin Transformer (SRCL)	15 million	Tile-level (256×256)	TCGA, PAIP
REMEDIS [10]	ResNet 152×2 (SimCLR)	50 million	Tile-level (256×256)	TCGA
Prov-GigaPath	**ViT (DINOv2) LongNet (MAE)**	**1.3 billion**	**Slide-level (up to $70K \times 70K$)**	**Providence**

Supplementary Table 1: **Overview of pathology image foundation models.** Comparison of pathology foundation models in terms of model architecture, data size, input size, and data source. *Prov-GigaPath* is the only one that models the whole slide during pretraining. Moreover, *Prov-GigaPath* is an open-weight model pretrained from real-world patient data.

Task	***Prov-GigaPath***	HIPT	CtransPath	REMEDIS	p-value
NSCLC Typing	**0.756 ± 0.010**	0.657 ± 0.013	0.732 ± 0.014	0.570 ± 0.015	0.065
BRCA Typing	**0.899 ± 0.015**	0.823 ± 0.027	0.895 ± 0.017	0.838 ± 0.025	0.539
RCC Typing	**0.953 ± 0.007*****	0.900 ± 0.012	0.919 ± 0.011	0.804 ± 0.025	0.001
COADREAD Typing	**0.834 ± 0.009***	0.774 ± 0.013	0.799 ± 0.011	0.724 ± 0.018	0.014
HB Typing	**0.905 ± 0.015***	0.857 ± 0.023	0.858 ± 0.016	0.849 ± 0.013	0.010
DIFG Typing	**0.970 ± 0.003****	0.943 ± 0.008	0.945 ± 0.007	0.885 ± 0.011	0.005
OVT Typing	**0.978 ± 0.003*****	0.946 ± 0.006	0.942 ± 0.007	0.834 ± 0.022	0.001
CNS Typing	**0.956 ± 0.003*****	0.902 ± 0.006	0.922 ± 0.005	0.808 ± 0.019	0.010
EGC Typing	**0.874 ± 0.011**	0.857 ± 0.011	0.868 ± 0.013	0.832 ± 0.013	0.423
Pan EGFR	**0.675 ± 0.011*****	0.637 ± 0.009	0.537 ± 0.006	0.613 ± 0.008	0.001
Pan FAT1	0.648 ± 0.008	**0.650 ± 0.005**	0.646 ± 0.007	0.638 ± 0.006	0.652
Pan KRAS	**0.775 ± 0.004*****	0.700 ± 0.006	0.642 ± 0.010	0.691 ± 0.009	0.001
Pan LRP1B	**0.678 ± 0.006*****	0.644 ± 0.008	0.639 ± 0.009	0.638 ± 0.008	0.001
Pan TP53	**0.724 ± 0.006*****	0.653 ± 0.008	0.615 ± 0.006	0.679 ± 0.009	0.001
LUAD EGFR	**0.543 ± 0.011***	0.494 ± 0.012	0.510 ± 0.012	0.511 ± 0.011	0.032
LUAD FAT1	**0.712 ± 0.012***	0.682 ± 0.014	0.688 ± 0.009	0.671 ± 0.019	0.024
LUAD KRAS	**0.547 ± 0.008***	0.536 ± 0.008	0.508 ± 0.014	0.532 ± 0.010	0.042
LUAD LRP1B	**0.688 ± 0.014**	0.655 ± 0.012	0.683 ± 0.013	0.651 ± 0.014	0.348
LUAD TP53	**0.638 ± 0.015***	0.612 ± 0.012	0.614 ± 0.012	0.607 ± 0.016	0.042
LUAD EGFR (TCGA)	**0.766 ± 0.012****	0.606 ± 0.015	0.541 ± 0.016	0.619 ± 0.014	0.002
LUAD FAT1 (TCGA)	**0.552 ± 0.021**	0.466 ± 0.012	0.503 ± 0.015	0.523 ± 0.032	0.216
LUAD KRAS (TCGA)	**0.610 ± 0.012**	0.596 ± 0.010	0.472 ± 0.014	0.578 ± 0.006	0.188
LUAD LRP1B (TCGA)	**0.598 ± 0.014****	0.553 ± 0.010	0.529 ± 0.012	0.553 ± 0.014	0.010
LUAD TP53 (TCGA)	**0.749 ± 0.011*****	0.679 ± 0.014	0.650 ± 0.016	0.702 ± 0.011	0.001
Pan 18-biomarkers	**0.649 ± 0.003*****	0.626 ± 0.003	0.600 ± 0.002	0.628 ± 0.003	0.001
Pan TMB	**0.708 ± 0.008**	0.657 ± 0.010	0.695 ± 0.008	0.676 ± 0.008	0.097

Supplementary Table 2: Table comparing *Prov-GigaPath* with state-of-the-art pathology foundation models on 26 tasks in pathomics and cancer subtyping using AUROC. $*$ indicates the significance level that *Prov-GigaPath* outperforms the best comparison approach on the specific task, with Wilcoxon test p-value$< 5 \times 10^{-2}$ for *, p-value$< 1 \times 10^{-2}$ for **, p-value$< 1 \times 10^{-3}$ for ***. The last column shows the p-value using the one-sided Wilcoxon test.

**Sex**	**% Patients**
Female	50.42%
Male	49.50%
None	0.08%

Supplementary Table 3: Table showing the sex distribution of patients in *Prov-Path*.

**Age**	**% Patients**
Below 11	0.21%
11-20	0.25%
21-30	1.09%
31-40	2.99%
41-50	8.70%
51-60	23.48%
61-70	32.37%
71-80	21.89%
81-90	13.43%
91-100	0.80%

Supplementary Table 4: Table showing the age distribution of patients in *Prov-Path*.

**Race**	**% Patients**
White or Caucasian	78.28%
Asian	4.31%
Black or African American	1.83%
American Indian or Alaska Native	0.76%
Native Hawaiian or Other Pacific Islander	0.33%
Unknown	8.20%
Patient Refused	1.97%
Other	4.32%

Supplementary Table 5: Table showing the self-reported ethnicity distribution of patients in *Prov-Path*.

Mutations	% Patients (Pan-cancer)	% Patients (LUAD)
TMB High	18.85%	27.09%
TMB Low	81.15%	72.91%
CD274 High	19.42%	27.95%
CD274 Low	35.09%	31.72%
TP53	47.18%	37.14%
LRP1B	16.78%	17.81%
KRAS	24.82%	40.48%
APC	15.12%	8.91%
KMT2D	12.48%	8.04%
FAT1	12.10%	11.30%
SPTA1	11.70%	11.30%
ZFHX3	11.63%	9.27%
KMT2C	10.88%	8.33%
EGFR	12.06%	25.92%
ARID1A	11.68%	9.78%
PIK3CA	13.15%	6.23%
PRKDC	10.77%	8.62%
NOTCH1	9.23%	5.94%
ATM	10.62%	12.53%
KMT2A	8.06%	7.68%
ROS1	9.10%	6.81%

Supplementary Table 6: Table showing the mutation rates of patients in *Prov-Path*.

**Gene mutation prediction**	**Mutations**	**Number of slides**
Pan 5-Gene	EGFR	545
	FAT1	462
	KRAS	1008
	LRP1B	639
	TP53	1774
LUAD 5-Gene	EGFR	227
	FAT1	94
	KRAS	375
	LRP1B	146
	TP53	357
Pan 18-biomarker	CD274	1975
	TP53	1774
	LRP1B	639
	KRAS	1008
	APC	576
	KMT2D	465
	FAT1	462
	SPTA1	447
	ZFHX3	425
	KMT2C	398
	EGFR	545
	ARID1A	433
	PIK3CA	496
	PRKDC	391
	NOTCH1	371
	ATM	412
	KMT2A	299
	ROS1	335
LUAD 5-Gene (TCGA)	EGFR	188
	FAT1	124
	KRAS	335
	LRP1B	423
	TP53	618
Pan TMB	High TMB	606
	Low TMB	2525

Supplementary Table 7: Table showing the number of slides for each class in each gene mutation prediction task. The first column represents 6 mutation prediction tasks, including pan-cancer and LUAD-specific 5-gene mutation predictions, Pan-cancer 18-biomarker prediction, LUAD-specific 5-gene mutation prediction on TCGA, and pan-cancer tumor burden prediction. The second column is the list of classes for each task and the third column is the number of slides within each class.

Cancer subtyping tasks	OncoTree code	Number of slides
NSCLC<br>(Non-Small Cell Lung Cancer)	LUAD (Lung Adenocarcinoma)<br>LUSC (Lung Squamous Cell Carcinoma)	685<br>315
BRCA<br>(Invasive Breast Carcinoma)	IDC (Breast Invasive Ductal Carcinoma)<br>ILC (Breast Invasive Lobular Carcinoma)	1181<br>57
RCC<br>(Renal Cell Carcinoma)	CCRCC (Renal Clear Cell Carcinoma)<br>PRCC (Papillary Renal Cell Carcinoma)<br>CHRCC (Chromophobe Renal Cell Carcinoma)	601<br>55<br>84
DIFG<br>(Diffuse Glioma)	GBM (Glioblastoma Multiforme)<br>ODG (Oligodendroglioma)<br>AODG (Anaplastic Oligodendroglioma)<br>HGGNOS (High-Grade Glioma, NOS)<br>AASTR (Anaplastic Astrocytoma)	68<br>384<br>30<br>27<br>21
COADREAD<br>(Colorectal Adenocarcinoma)	COAD (Colon Adenocarcinoma)<br>READ (Rectal Adenocarcinoma)	779<br>221
HB<br>(Hepatobiliary)	CHOL (Cholangiocarcinoma)<br>HCC (Hepatocellular Carcinoma)	74<br>137
CNS<br>(Central Nervous System)	ATM (Atypical Meningioma)<br>MNG (Meningioma)	862<br>517
EGC<br>(Esophagogastric<br>Adenocarcinoma)	ESCA (Esophageal Adenocarcinoma)<br>STAD (Stomach Adenocarcinoma)<br>GEJ (Adenocarcinoma of<br>the Gastroesophageal Junction)	336<br>43<br>102
OVT<br>(Ovarian Epithelial Tumor)	CCOV (Clear Cell Ovarian Cancer)<br>EOV (Endometrioid Ovarian Cancer)<br>HGSOC (High-Grade Serous Ovarian Cancer)<br>LGSOC (Low-Grade Serous Ovarian Cancer)<br>MOV (Mucinous Ovarian Cancer)<br>OCS (Ovarian Carcinosarcoma/<br>Malignant Mixed Mesodermal Tumor)	56<br>75<br>230<br>59<br>22<br>117

Supplementary Table 8: Table showing the number of slides for each class in each cancer subtyping task. The first column represents nine cancer subtyping tasks and the second column denotes the classes represented by OncoTree codes. The third column is the number of slides of each OncoTree code.

Tasks	# of training patients	# of validation patients	# of test patients
NSCLC typing	498	94	180
BRCA typing	161	63	96
RCC typing	90	39	54
DIFG typing	25	19	22
COADREAD typing	434	90	169
HB typing	56	13	25
CNS typing	91	61	73
EGC typing	75	25	38
OVT typing	47	18	26
LUAD mutation prediction	688	98	198
Pan-cancer mutation prediction	2,935	419	840
Pan-cancer TMB prediction	2,191	313	627

Supplementary Table 9: Table showing the number of patients in the training, validation, and test set across different tasks in *Prov-Path*.

References

[1] Chen, R. J. *et al.* Scaling vision transformers to gigapixel images via hierarchical self-supervised learning. In *Proceedings of the IEEE/CVF Conference on Computer Vision and Pattern Recognition*, 16144–16155 (2022).

[2] Oquab, M. *et al.* DINOv2: Learning robust visual features without supervision. *arXiv preprint arXiv:2304.07193* (2023).

[3] Caron, M. *et al.* Emerging properties in self-supervised vision transformers. In *Proceedings of the IEEE/CVF International Conference on Computer Vision*, 9650–9660 (2021).

[4] Zhou, J. *et al.* Image BERT Pre-training with Online Tokenizer. In *International Conference on Learning Representations* (2021).

[5] Caron, M. *et al.* Unsupervised learning of visual features by contrasting cluster assignments. *Advances in Neural Information Processing Systems* **33**, 9912–9924 (2020).

[6] Ding, J. *et al.* Longnet: Scaling transformers to 1,000,000,000 tokens. *arXiv preprint arXiv:2307.02486* (2023).

[7] Dao, T., Fu, D., Ermon, S., Rudra, A. & Ré, C. Flashattention: Fast and memory-efficient exact attention with io-awareness. *Advances in Neural Information Processing Systems* **35**, 16344–16359 (2022).

[8] He, K. *et al.* Masked autoencoders are scalable vision learners. In *Proceedings of the IEEE/CVF Conference on Computer Vision and Pattern Recognition*, 16000–16009 (2022).

[9] Wang, X. *et al.* Transformer-based unsupervised contrastive learning for histopathological image classification. *Medical Image Analysis* **81**, 102559 (2022).

[10] Azizi, S. *et al.* Robust and data-efficient generalization of self-supervised machine learning for diagnostic imaging. *Nature Biomedical Engineering* 1–24 (2023).
