# Supplementary material for: A whole-slide foundation model for digital pathology from real-world data
Source: Nature. 2024 May 22;630(8015):181–8. doi: 10.1038/s41586-024-07441-w (PMC11153137; doi:10.1038/s41586-024-07441-w)
Supplement: Supplementary file 2 — Reporting Summary [file 41586_2024_7441_MOESM2_ESM.pdf]

nature portfolio

Corresponding author(s): Hoifung Poon

Last updated by author(s): 2024/04/01

Reporting Summary

Nature Portfolio wishes to improve the reproducibility of the work that we publish. This form provides structure for consistency and transparency in reporting. For further information on Nature Portfolio policies, see our Editorial Policies and the Editorial Policy Checklist.

Statistics

For all statistical analyses, confirm that the following items are present in the figure legend, table legend, main text, or Methods section.

n/a	Confirmed	
☐	☒	The exact sample size (*n*) for each experimental group/condition, given as a discrete number and unit of measurement
☐	☒	A statement on whether measurements were taken from distinct samples or whether the same sample was measured repeatedly
☐	☒	The statistical test(s) used AND whether they are one- or two-sided<br>*Only common tests should be described solely by name; describe more complex techniques in the Methods section.*
☒	☐	A description of all covariates tested
☐	☒	A description of any assumptions or corrections, such as tests of normality and adjustment for multiple comparisons
☐	☒	A full description of the statistical parameters including central tendency (e.g. means) or other basic estimates (e.g. regression coefficient) AND variation (e.g. standard deviation) or associated estimates of uncertainty (e.g. confidence intervals)
☐	☒	For null hypothesis testing, the test statistic (e.g. *F*, *t*, *r*) with confidence intervals, effect sizes, degrees of freedom and *P* value noted<br>*Give P values as exact values whenever suitable.*
☒	☐	For Bayesian analysis, information on the choice of priors and Markov chain Monte Carlo settings
☒	☐	For hierarchical and complex designs, identification of the appropriate level for tests and full reporting of outcomes
☐	☒	Estimates of effect sizes (e.g. Cohen's *d*, Pearson's *r*), indicating how they were calculated

Our web collection on statistics for biologists contains articles on many of the points above.

Software and code

Policy information about availability of computer code

Data collection	We used the Databricks (runtime version 12.2 LTS) platform to collect the whole slide imaged from Providence. We used Microsoft SQL Azure (RTM) - 12.0.2000.8 and python==3.10 to collect histopathology findings, cancer staging, genomic mutation profiles, along with the associated pathology reports. For each whole slide image, we ran Otsu algorithm for tissue segmentation to filter background regions. For the pathology reports, we used GPT-3.5 provided by Azure OpenAI to extract clinical relevant information.
Data analysis	This work uses open source codebase and libraries to analyze the data. We used DINOv2 (https://github.com/facebookresearch/dinov2/tree/main) to pretrain the ViT tile encoder and OpenCLIP (https://github.com/mlfoundations/open_clip) to train the vision-language alignment model. For LongNet model, we used the implementation in torchscale==0.1.1. To install torchscale, we used the following public packages, including torch==2.0.0+cu117, torchvision==0.15.0+cu117, tensorboard==2.15.1, timm==0.9.12, xformers==0.0.18, einops==0.7.0, fairscale==0.4.13, huggingface-hub==0.19.4. We used scikit-learn==1.3.2, scipy==1.11.4 and numpy==1.24.1 to evaluate the model performance. We used matplotlib==3.3.0 to visualize the data.<br>All the codes to reproduce our experiments will be made public upon publication.

For manuscripts utilizing custom algorithms or software that are central to the research but not yet described in published literature, software must be made available to editors and reviewers. We strongly encourage code deposition in a community repository (e.g. GitHub). See the Nature Portfolio guidelines for submitting code & software for further information.

Data

Policy information about availability of data

All manuscripts must include a data availability statement. This statement should provide the following information, where applicable:

- Accession codes, unique identifiers, or web links for publicly available datasets
- A description of any restrictions on data availability
- For clinical datasets or third party data, please ensure that the statement adheres to our policy

The pathology imaging data used for the pretraining were created from oncology pathology slides at Providence. The associated clinical data used for fine-tuning and testing were obtained from the corresponding medical records. These proprietary data cannot be made publicly available. Researchers may obtain a de-identified test subset from Providence Health System by reasonable request and subject to local and national ethical approvals. To help researchers use our model, we provide a de-identified subset of our data at https://doi.org/10.5281/zenodo.10909616 and https://doi.org/10.5281/zenodo.10909922 for a few patients. We also collected publicly available TCGA whole slide images from NIH Genomic Data Commons Data Portal. The TCGA LUAD dataset, comprising whole pathology slides and labels, is available via the NIH Genomic Data Commons portal at https://portal.gdc.cancer.gov/projects/TCGA-LUAD.

Research involving human participants, their data, or biological material

Policy information about studies with human participants or human data. See also policy information about sex, gender (identity/presentation), and sexual orientation and race, ethnicity and racism.

Reporting on sex and gender	*Use the terms sex (biological attribute) and gender (shaped by social and cultural circumstances) carefully in order to avoid confusing both terms. Indicate if findings apply to only one sex or gender; describe whether sex and gender were considered in study design; whether sex and/or gender was determined based on self-reporting or assigned and methods used.*<br>*Provide in the source data disaggregated sex and gender data, where this information has been collected, and if consent has been obtained for sharing of individual-level data; provide overall numbers in this Reporting Summary. Please state if this information has not been collected.*<br>*Report sex- and gender-based analyses where performed, justify reasons for lack of sex- and gender-based analysis.*
Reporting on race, ethnicity, or other socially relevant groupings	*Please specify the socially constructed or socially relevant categorization variable(s) used in your manuscript and explain why they were used. Please note that such variables should not be used as proxies for other socially constructed/relevant variables (for example, race or ethnicity should not be used as a proxy for socioeconomic status).*<br>*Provide clear definitions of the relevant terms used, how they were provided (by the participants/respondents, the researchers, or third parties), and the method(s) used to classify people into the different categories (e.g. self-report, census or administrative data, social media data, etc.)*<br>*Please provide details about how you controlled for confounding variables in your analyses.*
Population characteristics	*Describe the covariate-relevant population characteristics of the human research participants (e.g. age, genotypic information, past and current diagnosis and treatment categories). If you filled out the behavioural & social sciences study design questions and have nothing to add here, write "See above."*
Recruitment	*Describe how participants were recruited. Outline any potential self-selection bias or other biases that may be present and how these are likely to impact results.*
Ethics oversight	*Identify the organization(s) that approved the study protocol.*

Note that full information on the approval of the study protocol must also be provided in the manuscript.

Field-specific reporting

Please select the one below that is the best fit for your research. If you are not sure, read the appropriate sections before making your selection.

☒ Life sciences ☐ Behavioural & social sciences ☐ Ecological, evolutionary & environmental sciences

For a reference copy of the document with all sections, see nature.com/documents/nr-reporting-summary-flat.pdf

Life sciences study design

All studies must disclose on these points even when the disclosure is negative.

Sample size	During the pretraining of our model, we used all 1,384,860,229 tiles in 171,189 pathology slides from 30,000 patients collected in the Providence health network comprising 28 cancer centers. For finetuning, we collected patients with the cancer that we investigate. Each pathology slide is a slide sample and each patient is a patient sample. Each patient can have several slides. When performing mutation prediction, we selected the largest slide for each patient to analyze. The sample size was determined by all the samples that were collected by August, 2023.
Data exclusions	We identified tiles that don't have a substantial tissue occupancy as the background area and filter them out from pretraining and finetuning.
Replication	Across all 26 tasks, we ran 10-fold cross-validation with 10 different seeds to determine whether the improvement of our model is significant

Replication	compared to baseline approaches.
Randomization	When doing the subtyping tasks and mutation prediction tasks, we randomly split the finetuning dataset into 7:1:2 train/validation/test splits. The hyperparameter was chosen based on accuracy on the validation set.
Blinding	During the test, the researchers were blinded to the group allocation.

Reporting for specific materials, systems and methods

We require information from authors about some types of materials, experimental systems and methods used in many studies. Here, indicate whether each material, system or method listed is relevant to your study. If you are not sure if a list item applies to your research, read the appropriate section before selecting a response.

Materials & experimental systems

n/a	Involved in the study
☒	☐ Antibodies
☒	☐ Eukaryotic cell lines
☒	☐ Palaeontology and archaeology
☒	☐ Animals and other organisms
☒	☐ Clinical data
☒	☐ Dual use research of concern
☒	☐ Plants

Methods

n/a	Involved in the study
☒	☐ ChIP-seq
☒	☐ Flow cytometry
☒	☐ MRI-based neuroimaging

Plants

Seed stocks	*Report on the source of all seed stocks or other plant material used. If applicable, state the seed stock centre and catalogue number. If plant specimens were collected from the field, describe the collection location, date and sampling procedures.*
Novel plant genotypes	*Describe the methods by which all novel plant genotypes were produced. This includes those generated by transgenic approaches, gene editing, chemical/radiation-based mutagenesis and hybridization. For transgenic lines, describe the transformation method, the number of independent lines analyzed and the generation upon which experiments were performed. For gene-edited lines, describe the editor used, the endogenous sequence targeted for editing, the targeting guide RNA sequence (if applicable) and how the editor was applied.*
Authentication	*Describe any authentication procedures for each seed stock used or novel genotype generated. Describe any experiments used to assess the effect of a mutation and, where applicable, how potential secondary effects (e.g. second site T-DNA insertions, mosiacism, off-target gene editing) were examined.*
